# A Fluorogenic Hydrazino‐Pictet–Spengler Reaction in Live Cells

**DOI:** 10.1002/anie.202510959

**Published:** 2025-08-25

**Authors:** Kaleena Basran, Nathan W. Luedtke

**Affiliations:** ^1^ Department of Chemistry McGill University 801 Sherbrooke St. West Montréal Québec H3A 0B8 Canada; ^2^ Department of Pharmacology and Therapeutics McGill University 3655 Prom. Sir William Osler Montréal Québec H3G 1Y6 Canada

**Keywords:** Aldehydes, Bioimaging, Fluorogenic reactions, Lipids, Oxidative stress

## Abstract

Lipid peroxidation generates diverse aldehydic lipids associated with oxidative stress and signaling, yet methodologies capable of their detection in live cells are currently lacking. Here, we report a fluorogenic hydrazino‐Pictet–Spengler (HIPS) probe for lipidic aldehydes “FLipA‐HIPS” that generates bright, wash‐free fluorescent reaction products in vitro and in cellulo. FLipA‐HIPS is essentially nonfluorescent but reacts with aldehydes to give an environmentally sensitive fluorophore with a higher quantum yield in SDS micelles as compared to aqueous buffer alone. FLipA‐HIPS reacts with lipidic aldehydes to give 10‐ to 25‐fold higher fluorescence intensities than reactions containing either short‐chain aliphatic or aromatic aldehydes in aqueous buffer. Confocal microscopy shows that live cells pretreated with potassium bromate or the aldehydic phospholipid “POVPC” display rapid and selective FLipA‐HIPS staining that co‐localizes with the endoplasmic reticulum (ER), consistent with the ER's known role in accumulating lipidic aldehydes. These results demonstrate that HIPS reactions can exhibit useful fluorogenic properties for aldehyde biosensing applications in live cells.

## Graphical abstract

A fluorogenic hydrazino‐Pictet–Spengler (HIPS) ligation reaction is used to label lipid‐derived aldehydes under wash‐free conditions in live cells. This innovative technique enables wash‐free imaging within biological systems, offering invaluable insights into the extent and localization of oxidative stress.

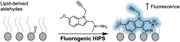

Fluorogenic “click” reactions provide powerful tools for characterizing the Golgi‐ER,^[^ ^1^ ^]^ as well as monitoring phospholipid dynamics,^[^ ^2^, ^3^ ^]^ lipid signaling,^[^ ^4^, ^5^, ^6^, ^7^ ^]^ and post‐translational protein modifications.^[^ ^8^, ^9^ ^]^ The mechanisms of these fluorogenic reactions are typically mediated by the bioorthogonal functional group, with fluorophore brightness increasing upon consumption of a conjugated azide,^[^ ^10^, ^11^ ^]^ alkyne,^[^ ^12^, ^13^ ^]^ phosphine,^[^ ^14^ ^]^ or tetrazine.^[^ ^15^, ^16^ ^]^ By comparison, fluorogenic click reactions that generate a fluorophore as the product remain far less explored,^[^ ^17^, ^18^, ^19^ ^]^ particularly in the context of lipid labeling.

Condensation of aldehydes or ketones with α‐nucleophiles was one of the earliest bioorthogonal chemical reactions evaluated for bioconjugation applications by Mahal and co‐workers.^[^ ^20^ ^]^ Efforts to develop fluorescence‐based strategies to detect cellular aldehydes now include hydrazino‐ or aminooxy‐probes to conjugate a diverse range of probes to biomolecules^[^ ^21^ ^]^ including proteins^[^ ^22^, ^23^, ^24^ ^]^ and glycoproteins,^[^ ^25^, ^26^ ^]^ multifunctional bioconstructs,^[^ ^27^ ^]^ DNA abasic sites,^[^ ^28^, ^29^, ^30^, ^31^, ^32^ ^]^ and simple aliphatic aldehydes.^[^ ^33^, ^34^ ^]^ These approaches are typically limited by competing reversible hydrolysis and irreversible enzymatic aldehyde oxidation reactions.^[^ ^35^, ^36^ ^]^ To address these challenges, Agarwal and coworkers reported Pictet–Spengler ligations yielding hydrolytically stable protein conjugates.^[^ ^37^ ^]^ This ligation strategy was expanded to the hydrazino‐Pictet–Spengler (HIPS) conjugation for the generation of stable conjugates at formylglycine sites in proteins.^[^ ^38^, ^39^, ^40^, ^41^ ^]^ Applications of this approach have included the production of antibody‐drug candidates^[^ ^42^ ^]^ and the analysis of aldehydic DNA by pull‐down and sequencing techniques.^[^ ^43^ ^]^

Among biomolecules, polyunsaturated fatty acids (PUFAs) and their esters are the most readily oxidized by reactive oxygen species (ROS), resulting in formation of lipid hydroperoxides (LOOH) that break down to generate diverse aldehyde products (Figure 1a).^[^ ^44^ ^]^ As compared to modern, cycloaddition‐based click reactions, carbonyl condensation reactions were reported to exhibit limited selectivity in live cells due to the presence of endogenous, water‐soluble metabolites such as reducing sugars. Here, we show that lipidic aldehydes, by virtue of their hydrophobic properties, can serve as effective bioorthogonal functional groups for fluorogenic HIPS reactions in live cells. Here we introduce FLipA‐HIPS, a new fluorogenic probe that reacts with aldehydes to generate a tricyclic “push‐pull” fluorophore comprising a methoxybenzene ring fused to a diazinium ring system. The fluorescence intensities of the reaction products are highly sensitive to the identity of the aldehyde, exhibiting selectivity for lipophilic aldehydes over hydrophilic aldehydes in vitro and in live cells under wash‐free conditions (Figure 1b).

**Figure 1:**
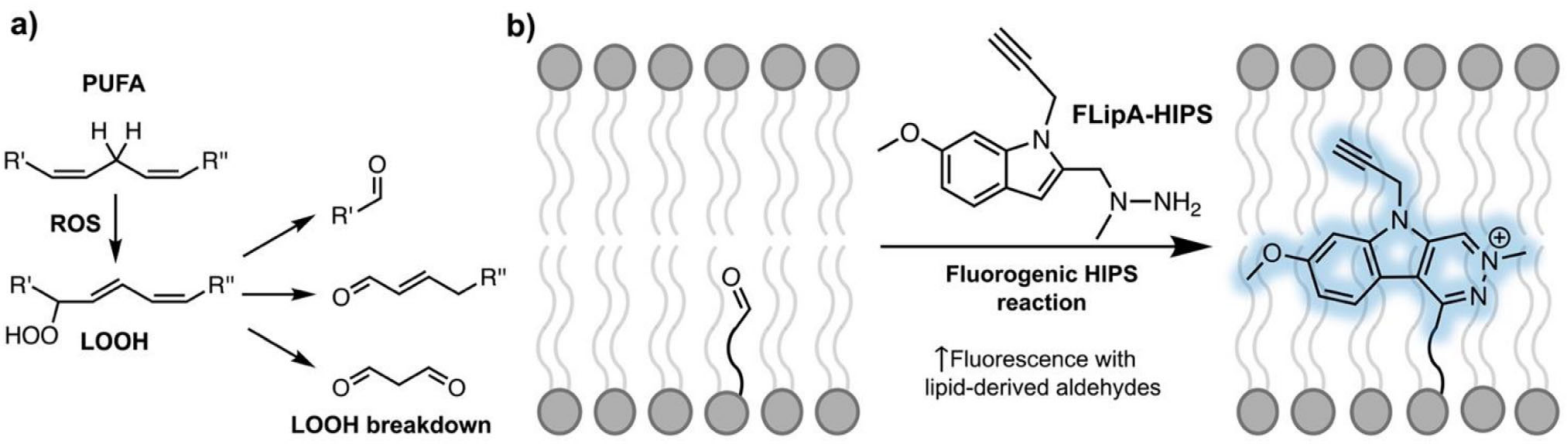
a) Lipid peroxidation of polyunsaturated fatty acids (PUFA) by reactive oxygen species (ROS) generates lipid hydroperoxides (LOOH) that break down into various aldehydic lipids.^[^ ^45^ ^]^ b) FLipA‐HIPS reacts with lipidic aldehydes to give bright, fluorescent products in vitro and in cellulo.

To synthesize FLipA‐HIPS, 6‐methoxyindole‐2‐carboxylic acid was reduced to 6‐methoxyindole‐2‐carbaldehyde **1** over three consecutive steps in an 86% yield. A propargyl group was introduced by treating **1** with an excess of sodium hydride followed by the dropwise addition of propargyl bromide to give **2** in 97% yield. In parallel, the Fmoc‐protected hydrazine, (9*H*‐fluoren‐9‐yl)methyl 2‐methylhydrazine‐1‐carboxylate **4**, was prepared according to literature procedures.^[^ ^43^ ^]^ Reductive amination between **2** and **4** with sodium triacetoxyborohydride yielded compound **3** in 74% yield. Fmoc‐deprotection by piperidine yielded FLipA‐HIPS in a 75% isolated yield (Scheme 1).

**Scheme 1:**
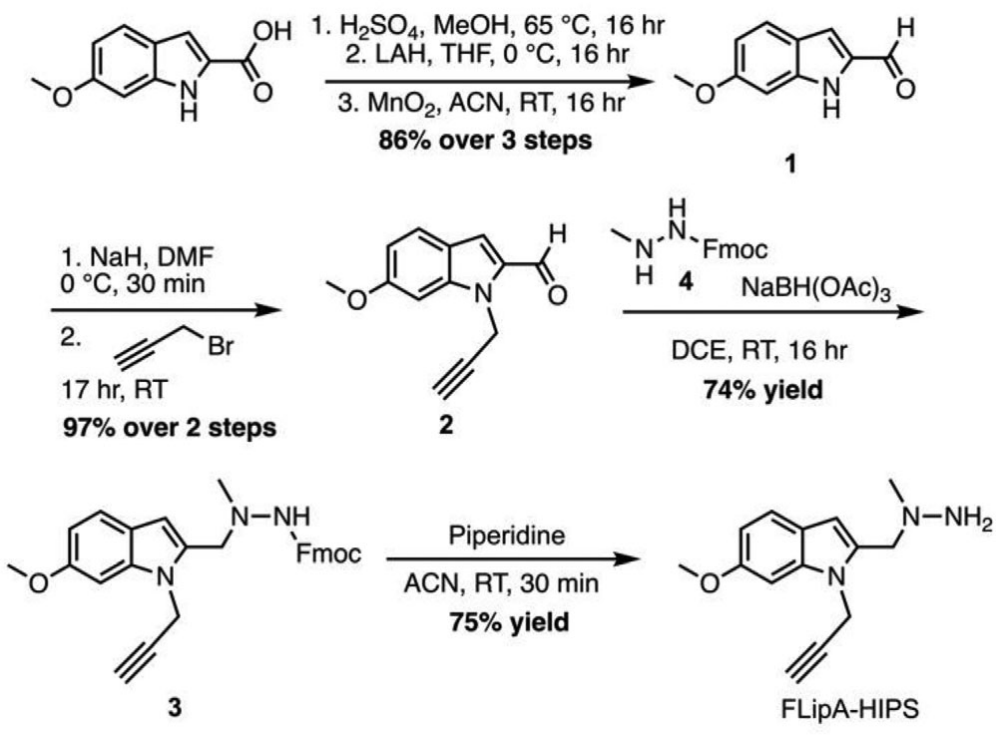
Synthesis of FLipA‐HIPS. See Supporting Information for synthetic procedures and characterizations. MeOH = methanol, LAH = lithium aluminum hydride, THF = tetrahydrofuran, ACN = acetonitrile, DMF = *N,N*‐dimethylformamide, DCE = dichloroethane, Fmoc = fluorenylmethoxycarbonyl, and RT = room temperature.

To investigate the fluorogenic potential of FLipA‐HIPS, it was reacted with acetaldehyde overnight in an open reaction vessel to yield diazinium **5** in an 82% crude yield according to ^1^H NMR (Figure 2a). The product was further purified by silica gel chromatography after treatment with methanesulfonic acid to yield the mesylate salt of **5** in 29% yield over three steps (see ESI†). Diazinium **5** mesylate exhibited bright fluorescence and redshifted absorbance as compared to the starting material FLipA‐HIPS, which is essentially nonfluorescent upon excitation at 350 nm (Figure 2b). In acetonitrile, diazinium **5** exhibited an emission maximum (***λ***_max_) at 440 nm and fluorescence quantum yield (*Φ*) of 0.33 (Figure 2c). In water and 1X PBS (pH 7.4), a redshifted ***λ***_max _= 450 nm and lower *Φ* = 0.17–0.28 was observed, consistent with the typical properties of a “push‐pull” fluorophore.^[^ ^46^ ^]^

**Figure 2:**
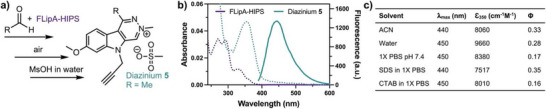
a) Reacting FLipA‐HIPS with acetaldehyde overnight in air followed by treatment with methanesulfonic acid (MsOH) yielded diazinium **5** as the mesylate salt (see ESI†). b) Absorbance and emission spectra of FLipA‐HIPS as compared to diazinium **5** (5 µM) in water containing 0.5% DMSO. c) Summary of photophysical properties for diazinium **5** mesylate salt under various conditions. Micelles were formed using either SDS = sodium dodecyl sulfate (38 mM) or CTAB = cetyltrimethylammonium bromide (1 or 38 mM) according to their critical micelle concentrations.^[^ ^47^ ^]^ For fluorescence emission spectra, excitation wavelength (*λ*_ex_) = 350 nm.

To further investigate the environmental sensitivity of diazinium **5**, its fluorescence properties were evaluated in aqueous buffer (1X PBS) upon addition of micelles composed of sodium dodecyl sulfate (SDS) or cetyltrimethylammonium bromide (CTAB). In the presence of anionic SDS micelles, which mimic biological membranes,^[^ ^48^ ^]^ diazinium **5** exhibited a blue‐shifted **λ**_max_ of 440 nm and enhanced fluorescence quantum yield (Φ) of 0.35 (Figure 2c). These values are similar to those of diazinium **5** mesylate in acetonitrile, suggesting that SDS micelles are capable of sequestering **5** from aqueous buffer. In contrast, the addition of cationic CTAB micelles produced no significant changes in fluorescence properties relative to 1X PBS, suggesting favorable electrostatic interactions between the anionic SDS head groups and the positively charged diazinium scaffold are important for its sequestration from water. These results suggest that the environmental sensitivity of diazinium **5** is well suited for phospholipid membranes (Figure 1b).

To evaluate its apparent second‐order rate constant, the fluorescence intensities of FLipA‐HIPS samples were monitored over time after the addition of 10‐ to 50‐fold excess acetaldehyde in aqueous 1 M sodium citrate (pH 5.0). The progress of each reaction was tracked by monitoring emission at 440 nm (Figure S1, ESI†). Using pseudo‐first‐order approximations, an apparent second‐order rate constant of *k*_app_ = 1.9 x 10^−2^ M^−1^s^−1^ was estimated. A comparable value has been reported for Pictet–Spengler coupling of aminooxy nucleophiles with peptides;^[^ ^49^ ^]^ however, competing background degradation of FLipA‐HIPS and the multistep formation of fluorescent products restrict interpretation of the kinetic data to relative rather than absolute measures.

To evaluate the scope of FLipA‐HIPS reactions, we screened a collection of aliphatic and aromatic aldehydes having different physical properties. A 12.5‐fold excess of each aldehyde (see Figure S2, ESI† for structures) was added to 20 µM of FLipA‐HIPS in 100 mM sodium citrate buffer (pH 5.0). The progress of each reaction was tracked by the emission at 450 nm (Figure 3). Remarkably, the medium and long‐chain lipidic aldehydes, nonanal and 1‐palmitoyl‐2‐(5‐oxovaleroyl)‐sn‐glycero‐3‐phosphorylcholine (POVPC), caused much larger increases in fluorescence as compared to all other aldehydes tested. Relative to acetaldehyde (ΔRFU defined as 1.0), POVPC elicited a 25‐fold higher fluorescence intensity, and nonanal a 10‐fold higher intensity. All other aldehydes exhibited more modest increases, ranging from 1.1‐ to 3.0‐fold higher than acetaldehyde after 3 h. A correlation was observed between the magnitude of fluorescence increases and aldehyde hydrophobicity based on calculated logP values. Aldehydes with positive calculated logP values ranging from 0.5 to 5.9 produced fluorescence increases ranging from 2.2‐ to 25.3‐fold, while those with negative calculated logP values gave similar fluorescence changes as acetaldehyde. The relative apparent rate of each reaction (*k′_rel_*) as compared to acetaldehyde (defined as 1.0) were only slightly lower for POVPC and nonanal (0.23 – 0.91), despite their tendency to self‐assemble into micellar structures in water.^[^ ^51^ ^]^ These hydrophobic reaction partners likely cause enhanced fluorescence changes by either acceleration of the reaction to give higher yields of fluorescent reaction products and/or by providing desolvated product environments as observed for SDS micelles (Figure 2c).

**Figure 3:**
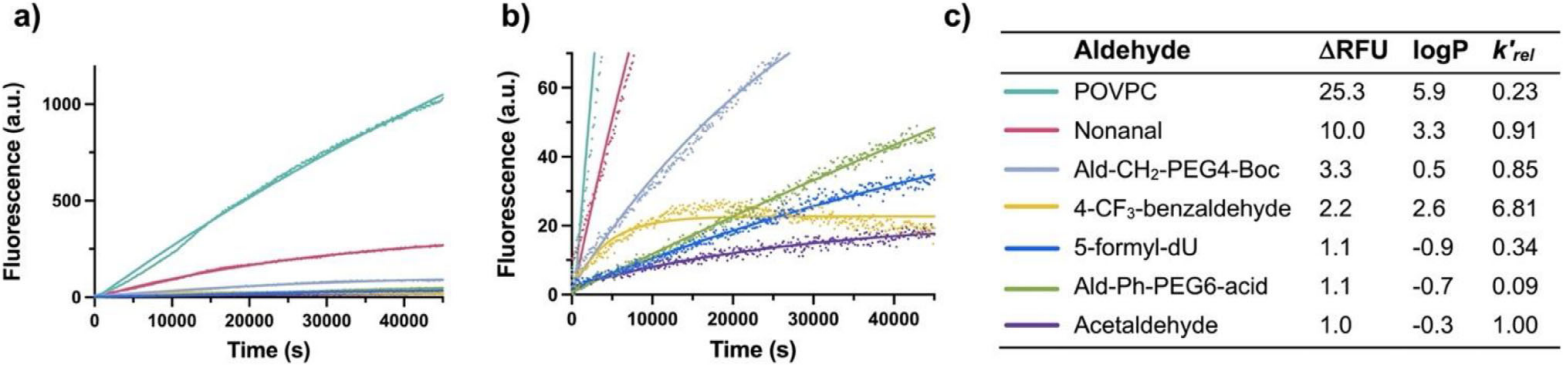
a) and b) Fluorescence changes (*λ*_ex_ = 350 nm) of FLipA‐HIPS (20 µM) following addition of various aldehydes (250 µM) in 100 mM sodium citrate buffer (pH 5.0). Fluorescence intensities were corrected by the background fluorescence changes of FLipA‐HIPS alone. See ESI for raw data in triplicate. c) Relative changes in fluorescence (ΔRFU) of each reaction after 3 h, octanol‐water partition coefficient (logP) values as computed by XLogP3 3.0.^[^ ^50^ ^]^ and relative apparent rate (*k'_rel_*) with respect to acetaldehyde defined as 1.0.

To evaluate the ability of FLipA‐HIPS to detect cellular aldehydes, we treated HeLa cells with potassium bromate (KBrO_3_), which is known to trigger lipid peroxidation and other types of oxidative damage.^[^ ^52^ ^]^ HeLa cell cultures were treated with 5 mM KBrO_3_ for 17 h, washed, and subsequently incubated with 10 µM FLipA‐HIPS for 4 h at 37 °C. Confocal microscopy revealed staining in KBrO_3_‐treated cells that was much brighter than cells receiving the FLipA‐HIPS probe but no KBrO_3_ (Figure 4a). The staining patterns were consistent with the endoplasmic reticulum (ER), which is largely responsible for the biosynthesis of lipids,^[^ ^53^ ^]^ and is often implicated in lipid peroxidation.^[^ ^54^ ^]^ To test this hypothesis, we repeated the experiment and added “BODIPY FL Glibenclamide” as an ER‐selective counter stain.^[^ ^55^ ^]^ Two‐color confocal microscopy revealed extensive co‐localization of the FLipA‐HIPS and ER signals in the KBrO_3_‐treated HeLa cells, with a Pearson's correlation coefficient (PCC) of 0.730 (Figure 4b). Similar results were obtained with U2OS cells (PCC = 0.677), which exhibited more punctate ER staining patterns and stronger FLipA‐HIPS reaction signals in the absence of KBrO_3_ (Figure S3). KBrO_3_ treatment alone produced no detectable blue fluorescence in the cells (Figure S4). These results are consistent with the FLipA‐HIPS probe detecting lipid aldehyde products and/or protein‐aldehyde adducts introduced by lipid peroxidation in the ER.

**Figure 4:**
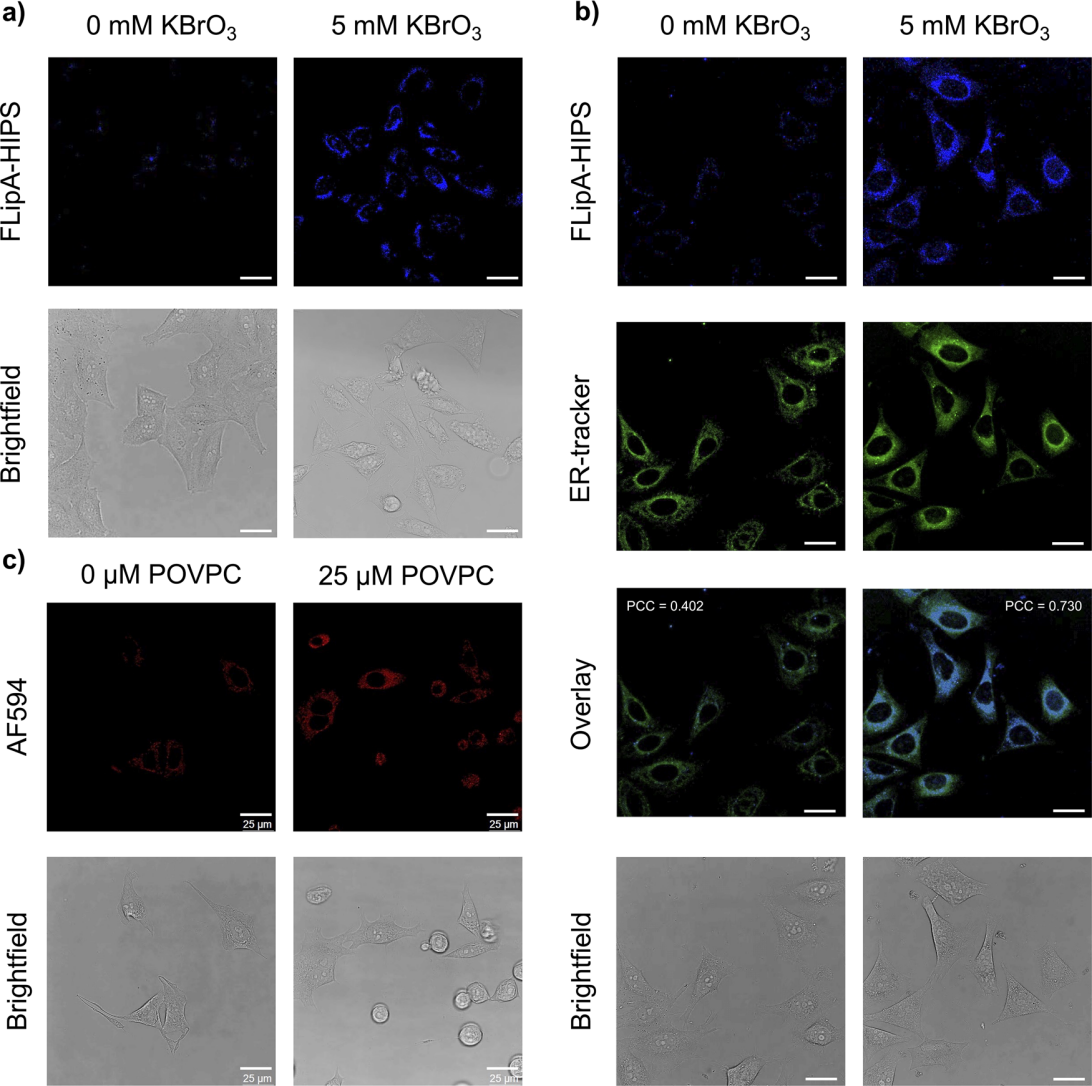
a) Wash‐free fluorescence imaging of HeLa cells treated with 5 mM KBrO_3_ for 17 h followed by 4 h of 10 µM FLipA‐HIPS at 37 °C. b) Following treatment with KBrO_3_ and FLipA‐HIPS, cells were washed and treated with 2 µM of BODIPY FL Glibenclamide as an ER‐tracker for 30 min at 37 °C before confocal microscopy. c) Fluorescence imaging of HeLa cells treated with 25 µM POVPC for 1 h followed by 2 h of 10 µM FLipA‐HIPS. Scale bars represent 25 µm. FLipA‐HIPS: *λ*_ex _= 405 nm, ER‐tracker: *λ*_ex _= 488 nm, and AF594: *λ*_ex _= 561 nm.

To evaluate the possibility that FLipA‐HIPS is reacting with aldehydes throughout the cell, but only those within the ER exhibit bright fluorescence, we performed a co‐localization experiment using the propargyl group. After fixing the treated cells with cold methanol, we performed a copper‐mediated azide‐alkyne cycloaddition (CuAAC) with AlexaFluor 647 (AF647) azide. As compared to live cells, the imaging quality was diminished and the signal distribution was different in the fixed cells, but a good overlap was observed between the fluorescence of the FLipA‐HIPS probe and the conjugated AF647 label with a PCC = 0.635 (Figure S5, ESI†). These results suggest that the innate fluorescence properties of the FLipA‐HIPS reaction products are sufficient for assessing their cellular localization. In addition to assessing the fluorescence staining patterns of fixed cells, the propargyl group of FLipA‐HIPS can enable “pull‐down” bioanalytical studies of tagged products.^[^ ^43^ ^]^

To further evaluate the selectivity of FLipA‐HIPS for lipidic aldehydes in live cells, we used FLipA‐HIPS to track the localization of the aldehydic phospholipid POVPC, a well‐established marker of oxidative lipid damage.^[^ ^56^ ^]^ HeLa cells were treated with 25 µM of POVPC for 1 h, followed by incubation with 10 µM of FLipA‐HIPS for 2 h without intermediate washing. Untreated cells showed substantially lower FLipA‐HIPS labeling as compared to those treated with POVPC (Figure S6, ESI†). The same ER‐selective staining patterns were observed in the live, POVPC‐treated cells as compared to those treated with potassium bromate. Co‐localization was also observed in fixed cells after CuAAC reactions with AlexaFluor 594 (AF594) azide, with an average PCC value of 0.778 ± 0.06 between FLipA‐HIPS fluorescence and that of AF594 (Figure 4c and S7, ESI†). The staining patterns in the live cells closely resemble those from previous studies, further supporting the ER as the major organelle to accumulate lipid peroxidation products including those derived from phosphatidylcholine.^[^ ^57^ ^]^

Membrane‐embedded lipidic aldehydes are known to disrupt bilayer packing and promote pore formation that can result in cell death at high levels.^[^ ^58^ ^]^ To address whether the observed FLipA‐HIPS staining patterns are being impacted by cell death, we used propidium iodide (PI) to assess membrane integrity. U2OS cells were treated with either 5 mM KBrO_3_ for 17 h or 25 µM POVPC for 1 h. POVPC‐treated cells were incubated with 10 µM FLipA‐HIPS for 2 h at 37 °C, while KBrO_3_‐treated cells were incubated with 10 µM FLipA‐HIPS for 4 h at 37 °C. Cells were then treated with 2.5 µg mL^−1^ of PI for 5 min prior to confocal microscopy of the live cells. In contrast to cells fixed with cold methanol before addition of PI, no detectable PI staining was observed in the cells treated with KBrO_3_, whereas ∼50% of cells treated with POVPC unexpectedly exhibited selective staining of the nucleoli rather than nuclei (Figure S8, ESI†). These results indicate that the live cells had not suffered catastrophic loss of membrane integrity upon addition of KBrO_3_ or POVPC under these conditions.

Hydrophobic cations, upon their addition to cells, often accumulate in the mitochondria.^[^ ^59^ ^]^ To evaluate potential co‐localization with mitochondria, living U2OS cells were treated with 25 µM POVPC for 1 h followed by 2 h of 10 µM FLipA‐HIPS at 37 °C. Cells were washed and treated with 300 nM MitoTracker Red (CMXRos) for 30 min at 37 °C. Confocal microscopy was used to image the live cells. In stark contrast to the results obtained using the ER tracker (PCC = 0.677, Figure S3, ESI†), we observed little‐to‐no co‐localization of the FLipA‐HIPS product(s) with MitoTracker Red in the live cells (PCC = 0.128, Figure S9, ESI†). The same cells were then washed with PBS and fixed using cold methanol prior to re‐imaging by confocal microscopy. Fixation resulted in a substantial increase in the co‐localization between the HIPS reaction products and MitoTracker Red (PCC = 0.395, Figure S9, ESI†). These findings suggest that FLipA‐HIPS reaction products form within ER‐associated vesicles in live cells and remain localized, redistributing to mitochondria only after cell fixation and permeabilization. Collectively, this underscores the importance of performing trafficking studies in live cells to avoid fixation‐induced artifacts.

To the best of our knowledge, this study provides the first reported examples of fluorogenic HIPS reactions. FLipA‐HIPS provides an effective tool to study the location and dynamics of lipid‐derived aldehydes using standard confocal microscopy, and suggests that lipidic aldehydes can be effective bioorthogonal reaction partners in live cells. Nonetheless, current drawbacks of this first‐generation probe include slow, multistep reaction kinetics and fluorescent products with absorbance maxima in the UV region (*λ*_max_ = 355 nm) which hinder certain flow cytometry and wide‐field microscopy applications. Furthermore, the limited chemical stability of FLipA‐HIPS necessitates its long‐term storage at −80 °C due to spontaneous degradation into products with low‐fluorescence intensities. The low intensities of the degradation products do not appear to interfere with live‐cell confocal microscopy experiments that further implicate the ER as the major organelle that accumulates lipid peroxidation products.^[^ ^57^ ^]^ The ability of cold methanol to re‐localize the FLipA‐HIPS reaction products to the mitochondria serves as a warning against overinterpretation of cellular co‐localization studies after common fixation procedures.

## Supporting Information

The authors have cited additional references within the Supporting Information.^[^ ^43^, ^60^, ^61^, ^62^ ^]^

## Conflict of Interests

The authors declare no conflict of interest.

## Supporting information



Supporting Information

## Data Availability

The data that support the findings of this study are available in the Supporting Information of this article.
