# Supplementary material for: A Fluorogenic Hydrazino‐Pictet–Spengler Reaction in Live Cells
Source: Angew Chem Int Ed Engl. 2025 Aug 25;64(40):e202510959. doi: 10.1002/anie.202510959 (PMC12462761; doi:10.1002/anie.202510959)
Supplement: Supplementary file 1 — Supporting Information [file ANIE-64-e202510959-s001.pdf]

A Fluorogenic Hydrazino-Pictet-Spengler Reaction in Live Cells

Kaleena Basran[a] and Nathan W. Luedtke*[a][b]

[a] Department of Chemistry, McGill University, Montreal, Quebec H3A 0B8, Canada

[b] Department of Pharmacology and Therapeutics, McGill University, Montreal, Quebec H3G 1Y6, Canada

Table of Contents

General Methods 2

Synthetic Procedures.......... 3

Supplemental Figures.......... 6

NMR Spectra 12

Raw Kinetic Data.......... 18

References.......... 20

General Methods

Materials, Methods, and Synthetic Procedures. Starting materials were obtained in the highest commercial grades and used without further purification. (9*H*-fluoren-9-yl)methyl 2-methylhydrazine-1-carboxylate **4** was synthesized according to previous reports.[1] 5-formyl-5',3'-diacetyl-2'-deoxyuridine was synthesized according to previous reports.[2] Reactions sensitive to moisture and/or air were carried out under an atmosphere of argon in anhydrous solvents and oven-dried glassware. Analytical thin-layer chromatography was performed on pre-coated 250 µm thick silica gel 60 F254 plates and visualized by ultraviolet light.

NMR spectra were recorded with a Bruker AVIII- 400 or AVIIIHD 500 (400 MHz for ^{1}H, 101 MHz for ^{13}C or 500 MHz for ^{1}H, 126 MHz for ^{13}C). ^{13}C spectra were measured with broadband proton decoupled. Chemical shifts (δ) are given in parts per million (ppm) with residual solvent peaks used as internal standards: d_6-DMSO (δH = 2.50 ppm), $CDCl_3$ (δH = 7.26 ppm), d_6-DMSO (δC = 40.5 ppm), or $CDCl_3$ (δC = 77.23 ppm). Coupling constants (*J*) are given in hertz (Hz). The following abbreviations were used to describe multiplicities: s = singlet, d = doublet, t = triplet, q = quartet, quint = quintet, sext = sextet, m = multiplet, dd = doublet-doublet, ddd = doublet-doublet-doublet, dt = doublet- triplet, dq = doublet-quartet, br = broad. High-resolution mass spectra were obtained on a Bruker MaXis high-resolution QTOF or a Thermo QExactive high-resolution Orbitrap. Masses are given as m/z.

Photophysical Properties of FLipA-HIPS. DMSO stock solutions of the FLipA-HIPS and the acetaldehyde trapped diazinium product **5** were prepared and stored at -20 °C. The samples were then thawed and diluted to an *OD* = 0.04 ± 0.01 (5 µM) at the most red-shifted absorbance maxima. For photophysical properties in micellular environments the concentration of FLipA-HIPS was increased to 20 µM with *OD* = 0.15 ± 0.01. All measurements were collected on a Molecular Devices SpectraMax M5 in a 1 cm path-length quartz cuvette. Quantum yields were calculated using the most red-shifted absorbance maxima of samples. Quinine hemisulfate (Φ_R = 0.546) in 0.5 M H_2SO_4 (n_R = 1.346) was used as a fluorescent standard. Quantum yields were calculated using the equation shown below:

$$\Phi = \Phi_R \frac{F}{F_R}\frac{A_R}{A}\frac{n^2}{n_R^2}$$

where Φ_R is the quantum yield of the fluorescent standard, *F* and F_R are the integrated emissions of the sample and reference respectively. *A* and A_R are the optical densities of the sample and reference respectively (both 0.04 ± 0.01). *n* and n_R are the refractive indexes of the sample and reference respectively.

Reaction Kinetics. Second order reaction rates between FLipA-HIPS and acetaldehyde were determined under pseudo-first-order conditions using a large excess of the aldehyde over the probe. Reaction progress was monitored by fluorescence emission at 440 nm (excitation: 350 nm, 365 nm cut-off filter) on a SpectraMax M5 plate reader (Molecular Devices, Sunnyvale, CA, USA) in a 1 cm quartz cuvette. Reaction mixtures contained 100 µM FLipA-HIPS with 1.0, 2.5 or 5.0 mM acetaldehyde (10 – 50 eq) in 1 M aq. sodium citrate buffer (pH 5.0, 0.1% DMSO). Fluorescence was recorded over 1.5 h at 2 min intervals. Control samples lacking acetaldehyde exhibited fluorescence increases which were subtracted from experimental data. The pseudo first-order rate constant of each reaction (*k*') was fitted from the background corrected data at each concentration using a mono-exponential non-linear regression $Y = Y_{max} * (1 - e^{(-k'*t)})$ where Y = fluorescence and t = time. The apparent second-order rate constant was determined from the slope of *k'* versus acetaldehyde concentration.

Relative apparent rates (k'_{rel}) for comparisons between various aldehydes were determined under pseudo-first-order conditions with an excess of each aldehyde. Reaction progress was monitored by fluorescence emission at 450 nm (excitation: 350 nm, 365 nm cut-off filter) using a SpectraMax M5 plate reader (Molecular Devices, Sunnyvale, CA, USA) in a 96-well plate. Reaction mixtures contained 20 µM FLipA-HIPS with 250 µM of aldehyde (12.5 eq) in 100 mM aq. sodium citrate buffer (pH 5.0, 0.5% DMSO). Fluorescence was recorded in triplicate over 17 h at 2 min intervals. Baseline drift was corrected using control wells containing 20 µM FLipA-HIPS without added aldehyde. The relative rate was measured by fitting the change in fluorescence over time to a non-linear regression considering the exponential plateau with a Y-intercept (Y_0) greater than -1, and using $Y = Y_{max} - (Y_{max} - Y_0) * e^{(-k'*t)}$ where Y = fluorescence, Y_{max} = maximum fluorescence, and t = time. The k'_{rel} values were reported with respect to the apparent rate of reaction of FLipA-HIPS with acetaldehyde.

Eukaryotic Cell Culture. Eukaryotic cells (HeLa or U2OS) were cultivated at 37 °C, 5% CO_2 in DMEM (Gibco) containing 4.5 g/L glucose, supplemented with 10% FBS (Gibco), 50,000 units Penicillin, and 50 mg Streptomycin per L (Sigma Aldrich), and 1% MEM non-essential amino acids (Sigma Aldrich) Cells were grown to confluency and passaged every 2 to 4 days using a Trypsin-EDTA solution (Sigma Aldrich). Cells were counted using a Bio-Rad TC20 Automated Cell Counter for the determination of seeding density.

Confocal Laser Scanning Microscopy (CLSM). Confocal Laser Scanning Microscopy (CLSM) was performed on Leica Stellaris 5 (Leica Microsystems) equipped with a HC PL APO 63x/1.40 OIL CS2 (FWD: 0.14 mm) objective. FLipA-HIPS was excited at 405 nm and emission was sampled between 415 and 485 nm. ER-Tracker Green (BODIPY® FL Glibenclamide) was excited at 488 nm and emission was sampled between 500 and 555 nm. MitoTracker Red (CMXRos) was excited at 561 nm and emission was sampled between 575 and 625 nm. Propidium iodide was excited at 561 nm and emission was sampled between 575 and 625 nm. AlexaFluor 594 was excited at 561 nm and emission was sampled between 575 and 625 nm. AlexaFluor 647 was excited at 638 nm and emission was sampled between 645 and 700 nm. HyD detectors were used. Image analysis was performed using Leica LAS AF Lite 2.6.3 (Leica

Microsystems). Colocalization analysis was conducted in Image-J software. Colocalization between two dyes was measured based on the Pearson correlation coefficient (PCC) using the JACoP and Colocalization Finder plugins.[3-4]

Potassium Bromate Induced Aldehyde Labelling. HeLa or U2OS cells were seeded in DMEM media in µ-slide 8-well chambers (ibidi®, cat-#: 80826) at densities of 5.0 × 10^4 – 5.5 × 10^4 cells per mL (10,000 cells per well) and allowed to settle overnight. They were aspirated and incubated with or without 5 mM of $KBrO_3$. After incubating for 16-18 h, cells were aspirated, washed with PBS, incubated with freshly diluted 10 µM FLipA-HIPS in media for 4 h at 37 °C. Cells remained living during this time and were imaged after the incubation with and without washing.

POVPC Labelling. HeLa or U2OS cells were seeded in DMEM media in µ-slide 8-well chambers (ibidi®, cat-#: 80826) at densities of 5.0 × 10^4 – 5.5 × 10^4 cells per mL (10,000 cells per well) and allowed to settle overnight. They were aspirated, cells were washed with serum-free media and then incubated in it with or without 25 µM of POVPC for 1 h. The cells were aspirated then immediately incubated with freshly diluted 10 µM FLipA-HIPS in media for 2 h at 37 °C. Cells remained living during this time and were imaged wash-free.

General Staining Procedures.
For endoplasmic reticulum staining, 2 µM of the ER-Tracker Green (BODIPY® FL Glibenclamide) was added immediately after aspirating the 10 µM FLipA-HIPS solution. The ER stain was incubated for 30 min at 37 °C before the cells were aspirated, washed with PBS and then the media was replaced with Invitrogen™ Live Cell Imaging Solution. The cells must be imaged while the cells are still living.

For mitochondria staining, 300 nM of MitoTracker Red was added and incubated for 30 min at 37 °C before the cells were aspirated, washed with PBS and then the media was replaced with Invitrogen™ Live Cell Imaging Solution. The cells could be imaged live or fixed.

For the copper-mediated azide-alkyne cycloaddition, the cells were fixed using cold methanol for 15 min in the freezer. The cells were washed with PBS before incubation with 2 mM THPTA, 1 mM $CuSO_4$, 10 µM azide dye (AF594 or AF647 azide), and 10 mM sodium ascorbate (added last) for 2 h at room temperature in the dark. Cells were washed with PBS three times and imaged using confocal microscopy. Cells can be left in PBS at 4 °C for up to 1 month.

For monitoring membrane integrity, a solution of 2.5 µg/mL of Propidium Iodide was added to the cells and incubated for 5 min at 37 °C. The cells were aspirated, washed with PBS and then the media was replaced with Invitrogen™ Live Cell Imaging Solution. The positive control of Propidium Iodide was prepared by seeding in DMEM media in µ-dish 35 mm (ibidi®, cat-#: 81218) at densities of 1.6 × 10^5 – 1.7 × 10^5 cells per mL (80,000 cells per well) and allowed to settle overnight. These cells were fixed using cold methanol for 15 min in the freezer. The cells were treated with a solution of 2.5 µg/mL of Propidium Iodide for 5 min, before washing with PBS and imaging using confocal microscopy.

Synthetic Procedures

6-methoxyindole-2-carbaldehyde (1)

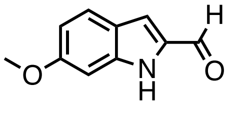

6-methoxyindole-2-carboxylic acid (362 mg, 1.89 mmol, 1.0 eq) was dissolved in 3.17 mL of methanol. The solution was purged with argon for 5 min. 0.48 mL of sulfuric acid was added dropwise. The reaction mixture was heated to 65 °C and refluxed overnight. The methanol was evaporated, and the reaction was quenched with $NaHCO_3$ (aq. sat.) until the bubbling subsided. The aqueous phase was extracted with EtOAc (2x15 mL). The combined organic phases were washed with brine (2x15 mL), dried over $MgSO_4$, and concentrated under reduced pressure. The crude brown-orange solid was dried on high vacuum and passed onto the next step without further purification.

(6-methoxy-1*H*-indol-2-yl)methanol (364 mg, 1.77 mmol, 1.0 eq) was dissolved in 17.7 mL of dry THF. The solution was purged with argon for 5 min and then cooled down to 0 °C. Lithium aluminium hydride (202 mg, 5.33 mmol, 3.0 eq) was added scoop wise, allowing for the bubbling to subside with each addition. The reaction mixture was stirred overnight allowing it to warm to room temperature. The reaction was quenched with water until the bubbling subsided. The mixture was diluted with 20 mL of EtOAc. This organic phase was decanted and washed with water (2x20 mL). The remaining aqueous phase was extracted with DCM (2x15 mL). The combined organic phases were dried over $MgSO_4$ and concentrated under reduced pressure. The crude brown solid was dried on high vacuum and dissolved in 9.8 mL of dry ACN. 800 mg of manganese dioxide (9.20 mmol, 5.2 eq) was added and the reaction was stirred overnight. The mixture was passed through celite, and the solvent was evaporated to yield 6-methoxyindole-2-carbaldehyde (284 mg, 1.62 mmol) as a dark brown solid in an 86% yield over 3 steps.

^{1}**H NMR** (400 MHz, $CDCl_3$) δ 9.76 (s, 1H), 7.62 (d, *J* = 8.8 Hz, 1H), 7.24 (d, *J* = 1.2 Hz, 1H), 6.90 (d, *J* = 2.3 Hz, 1H), 6.86 (dd, *J* = 8.8, 2.2 Hz, 1H), 3.90 (s, 3H). ^{13}**C NMR** (101 MHz, $CDCl_3$) δ 181.23, 160.43, 139.95, 135.49, 124.26, 121.81, 116.05, 113.48, 93.69, 55.53. **HR-ESI-MS (m/z):** $[M + Na]^+$ calc. for $C_{10}H_9NO_2Na$: 198.0525, found: 198.0532.

1-(Prop-2-yn-1-yl)-6-methoxy-1*H*-indole-2-carbaldehyde (2)

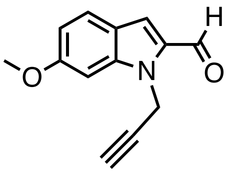

6-methoxyindole-2-carbaldehyde (44 mg, 0.25 mmol, 1.0 eq) was dissolved in 0.3 mL of dry DMF under argon. In a separate round bottom, 24 mg of sodium hydride (60% wt dispersed in oil, 0.27 mmol, 1.1eq) was dissolved in 1.4 mL of dry DMF at 0 °C under inert atmosphere. The indole solution was added dropwise to the sodium hydride round bottom. The reaction mixture was stirred for 30 min maintaining the temperature at 0 °C. 0.041 mL of propargyl bromide in toluene (9.2M, 0.38 mmol, 1.5 eq) was added slowly and the reaction was stirred overnight at room temperature. The reaction was quenched with 5 mL of NH_4Cl (aq. sat.). The aqueous phase was extracted with EtOAc (2x10 mL). The combined organic phases were washed with brine (2x10 mL), dried over $MgSO_4$, and concentrated under reduced pressure. The crude brown solid (53 mg, 0.24 mmol, 97% yield) was dried on high vacuum and passed onto the next step without further purification.

^{1}**H NMR** (400 MHz, $CDCl_3$) δ 9.74 (s, 1H), 7.59 (d, *J* = 9.4 Hz, 1H), 7.19 (s, 1H), 6.87 (dq, *J* = 5.2, 2.2 Hz, 2H), 5.42 (d, *J* = 2.5 Hz, 2H), 3.91 (s, 3H), 2.29 (t, *J* = 2.5 Hz, 1H).^{13}**C NMR** (101 MHz, $CDCl_3$) δ 181.59, 160.42, 141.65, 134.01, 124.51, 121.07, 119.45, 113.40, 92.24, 78.16, 72.54, 55.61, 33.92. **HR-ESI-MS (m/z):** $[M + H]^+$ calc. for $C_{13}H_{12}NO_2$: 214.08626, found: 214.08608.

(9*H*-fluoren-9-yl)methyl 2-methyl-2-((1-(prop-2-yn-1-yl)-6-methoxy-1*H*-indol-2-yl)methyl)hydrazine-1-carboxylate (3)

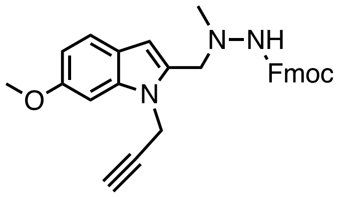

1-(Prop-2-yn-1-yl)-6-methoxy-1*H*-indole-2-carbaldehyde (55 mg, 0.25 mmol, 1.0 eq) and (9*H*-fluoren-9-yl)methyl 2-methylhydrazine-1-carboxylate (**4**, 76 mg, 0.28 mmol, 1.1 eq) were dissolved in 3.6 mL of dry dichloroethane and purged with argon. Sodium triacetoxyborohydride (106 mg, 0.50mmol, 2.0 eq) was added and the reaction was left stirring overnight. The reaction was quenched with 5 mL of $NaHCO_3$ (aq. sat.). The reaction was then extracted with DCM (2x10 mL) and the combined organic phases were washed with water (2x10 mL), dried over $MgSO_4$, and concentrated under reduced pressure. The crude product was purified by silica gel column chromatography (0 – 20% EtOAc in hexanes) to give a light brown solid (86 mg, 0.18mmol) in 74% yield.

^{1}**H NMR** (500 MHz, $CDCl_3$) δ 7.80 (d, *J* = 7.5 Hz, 2H), 7.60 – 7.48 (m, 2H), 7.44 (q, *J* = 7.0 Hz, 3H), 7.32 (s, 2H), 6.98 – 6.91 (m, 1H), 6.84 (dd, *J* = 8.6, 1.9 Hz, 1H), 6.34 (s, 1H), 6.04 (s, 1H), 5.21 (s, 2H), 4.44 (s, 2H), 4.07 (s, 2H), 3.91 (s, 3H), 2.66 (s, 3H), 2.29 (s, 1H). ^{13}**C NMR** (126 MHz, $CDCl_3$) δ 156.65, 143.87, 141.41, 138.17, 132.91, 127.81, 127.19, 125.08, 121.80, 121.38, 120.09, 109.86, 104.12, 102.57, 93.43, 79.18, 72.18, 66.62, 55.79, 55.30, 47.26, 44.26, 32.92. **HR-ESI-MS (m/z):** $[M + Na]^+$ calc. for $C_{29}H_{27}N_3O_3Na$: 488.19446, found: 488.19427.

2-((1-methylhydrazinyl)methyl)-1-(prop-2-yn-1-yl)-6-methoxy-1*H*-indole (FLipA-HIPS)

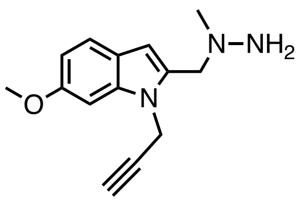

Compound **3** (84.6 mg, 0.182 mmol, 1.0 eq) was dissolved in 1.1 mL of dry ACN and purged with argon. Piperidine (0.18 mL, 1.82 mmol, 10.0 eq) was added and the reaction was stirred for 30 min at room temperature. The reaction was directly purified by silica gel column chromatography with a slow gradient (0 – 4% MeOH in DCM) to give a clear oil (33.5 mg, 0.137 mmol) in a 75% yield. It should be noted that this compound reacts rapidly with carbonyls such as acetone. Flame dried glassware should be used after the column. FLipA-HIPS should be stored in a -80 °C to preserve shelf life.

^{1}H NMR (500 MHz, $CDCl_3$) δ 7.44 (d, *J* = 8.6 Hz, 1H), 6.90 (d, *J* = 2.2 Hz, 1H), 6.80 (dd, *J* = 8.6, 2.2 Hz, 1H), 6.38 – 6.31 (m, 1H), 5.06 (d, *J* = 2.5 Hz, 2H), 3.89 (s, 3H), 3.79 (s, 2H), 2.52 (s, 3H), 2.26 (t, *J* = 2.5 Hz, 1H). **^{13}C NMR** (126 MHz, $CDCl_3$) δ 156.55, 138.06, 133.88, 121.80, 121.17, 109.73, 103.84, 93.36, 78.77, 72.11, 60.16, 55.76, 48.47, 32.91. **HR-ESI-MS (m/z):** $[M + H]^+$ calc. for $C_{14}H_{18}N_3O$: 244.14441, found: 244.14444.

7-methoxy-1,3-dimethyl-5-(prop-2-yn-1-yl)-5*H*-pyridazino[4,5-*b*]indol-3-ium (Diazinium 5)

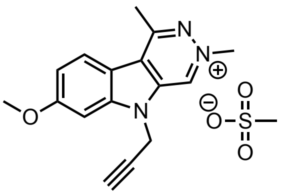

0.057 mL of acetaldehyde (1.02 mmol, 50.0 eq) was added to FLipA-HIPS (5.0 mg, 0.02 mmol, 1.0 eq). Reaction was stirred for overnight at ambient conditions. The acetaldehyde was evaporated, and the crude was passed through a silica gel column (20 – 100% EtOAc in hexanes followed by 100% MeOH) to give a yellow oil (6.0 mg, 0.02 mmol) with an 82% yield by ^{1}H NMR.

^{1}H NMR (500 MHz, DMSO) δ 10.57 (s, 1H), 8.40 (d, *J* = 9.0 Hz, 1H), 7.65 (d, *J* = 2.1 Hz, 1H), 7.31 (dd, *J* = 9.0, 2.2 Hz, 1H), 5.70 – 5.64 (m, 2H), 4.61 (s, 3H), 4.02 (s, 3H), 3.58 (t, *J* = 2.3 Hz, 1H), 3.13 (s, 3H).

0.4 mL of acetaldehyde (7.13 mmol, 86.6 eq) was added to FLipA-HIPS (20.0 mg, 82.3 µmol, 1.0 eq), the reaction was stirred for 30 min before 0.2 mL of water and the reaction was stirred overnight at ambient conditions. After 24 h, 1 mL of a 0.4% solution of methanesulfonic acid in water and stirred for 30 min. The reaction mixture was concentrated under reduced pressure and the brown oil was redissolved in minimal acetonitrile before purification by silica gel column chromatography (0 – 10% MeOH in DCM) to give a yellow oil (15.1 mg, 24.1 µmol) in a 29% yield.

^{1}H NMR (500 MHz, DMSO) δ 10.41 (s, 1H), 8.40 (d, *J* = 9.0 Hz, 1H), 7.66 (d, *J* = 2.1 Hz, 1H), 7.31 (dd, *J* = 9.0, 2.2 Hz, 1H), 5.65 (d, *J* = 2.3 Hz, 2H), 4.61 (s, 3H), 4.02 (s, 3H), 3.59 (t, *J* = 2.4 Hz, 1H), 3.14 (s, 3H). Counterion $CH_3SO_3^-$: δ 2.32 (s, 8H). **^{13}C NMR** (126 MHz, DMSO) δ 163.26, 154.48, 145.12, 136.13, 133.02, 125.79, 120.98, 115.18, 112.36, 94.54, 77.10, 77.09, 56.34, 51.12, 33.70, 20.53. **HR-ESI-MS (m/z):** $[M]^+$ calc. for $C_{16}H_{16}N_3O$: 266.1288, found: 266.1282.

Supplemental Figures

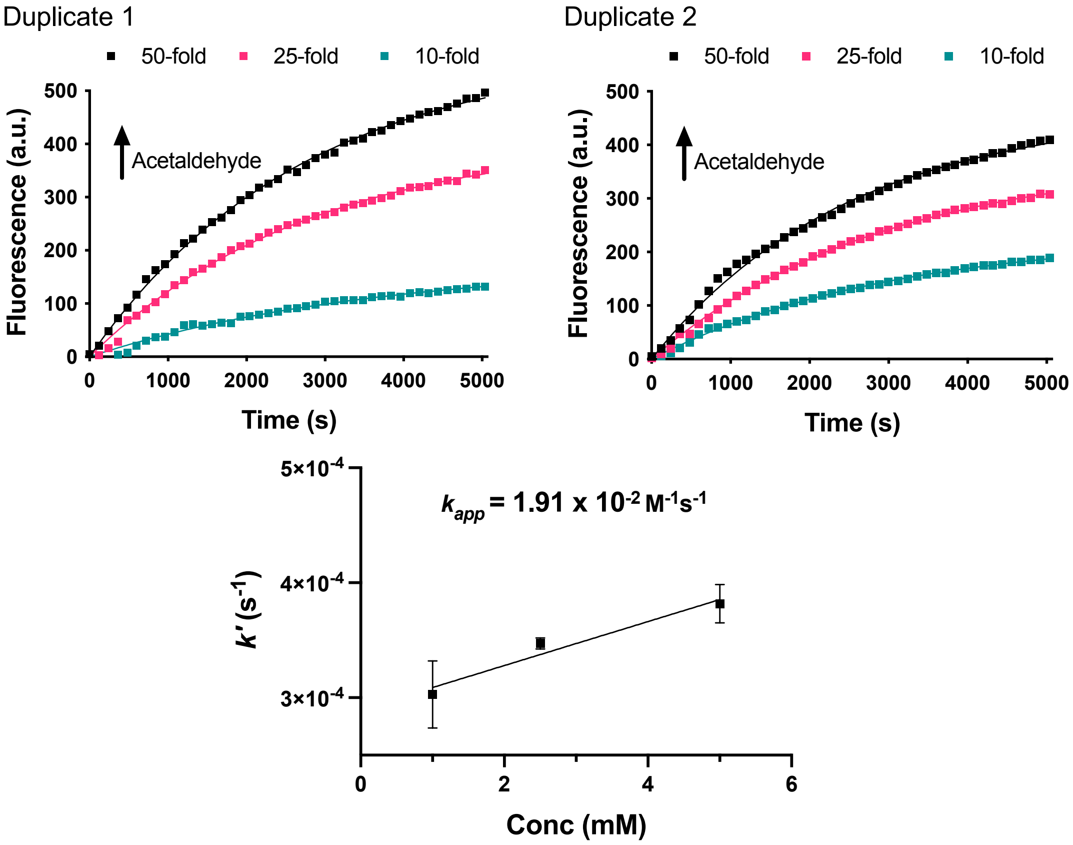

Figure S1. Kinetic analysis of FLipA-HIPS (100 µM) reacting with acetaldehyde (0 – 5 mM, 0 – 50 eq) in 1 M citrate buffer (pH 5.0). Fluorescence (a.u.) increase versus time upon mixing our probe with acetaldehyde. The data were corrected by the fluorescence increases exhibited by FLipA-HIPS under the same reaction conditions but in the absence of added aldehyde. Plot of the pseudo first-order rate constants (k') versus acetaldehyde concentrations. The slope yields the apparent rate constant k_{app}.

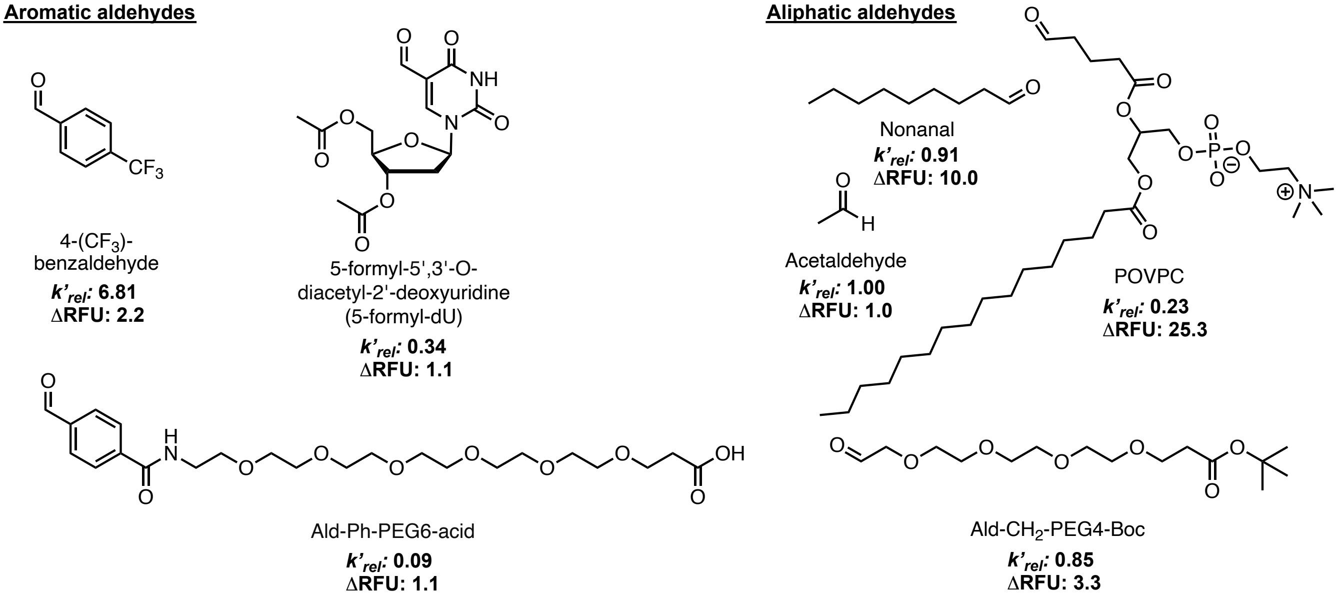

Figure S2. Structures of aliphatic and aromatic aldehydes used in this study. Relative apparent rates (k'_{rel}) with respect to acetaldehyde, and the changes in fluorescence (ΔRFU) after 3 h following background correction by subtracting the fluorescence of FLipA-HIPS only samples in 100 mM citrate buffer (pH 5.0, 0.5% DMSO). Acetaldehyde values are defined as 1. POVPC = 1-palmitoyl-2-(5-oxovaleroyl)-sn-glycero-3-phosphorylcholine.

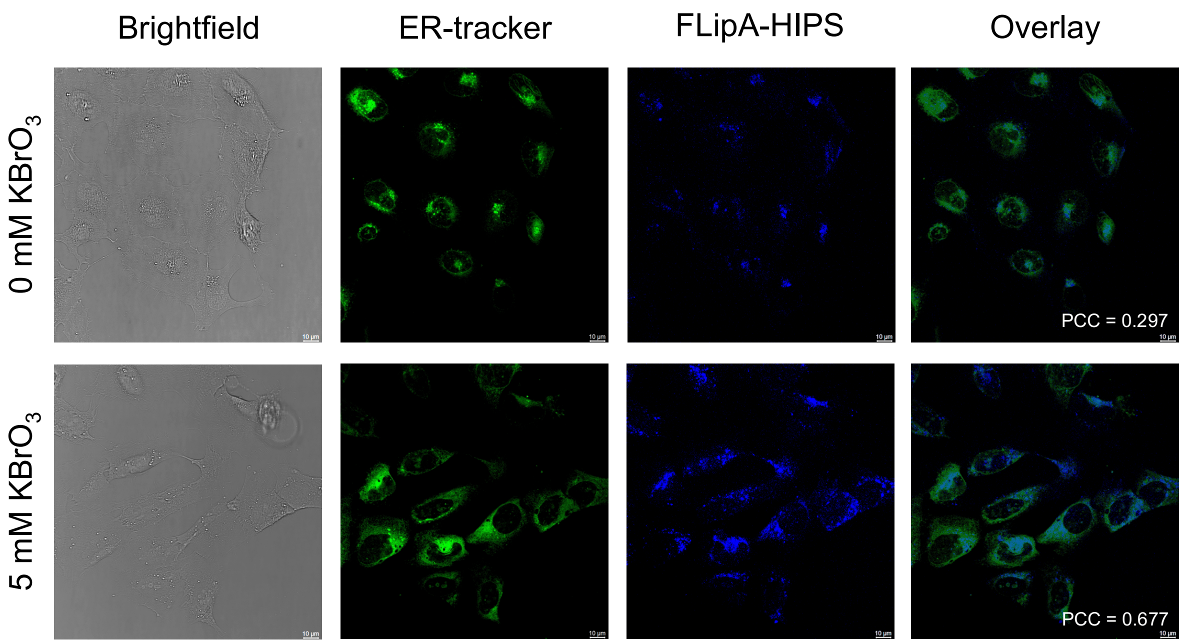

Figure S3. Fluorescence images of U2OS cells treated with or without 5 mM $KBrO_3$ for 17 h followed by treatment with 10 µM of FLipA-HIPS for 4 h at 37 °C. Cells were washed and treated with 2 µM of BODIPY FL Glibenclamide as an ER-tracker for 30 min at 37 °C before confocal microscopy. Scale bars represent 10 µm. FLipA-HIPS: λ_{ex} = 405 nm and ER-tracker: λ_{ex} = 488 nm.

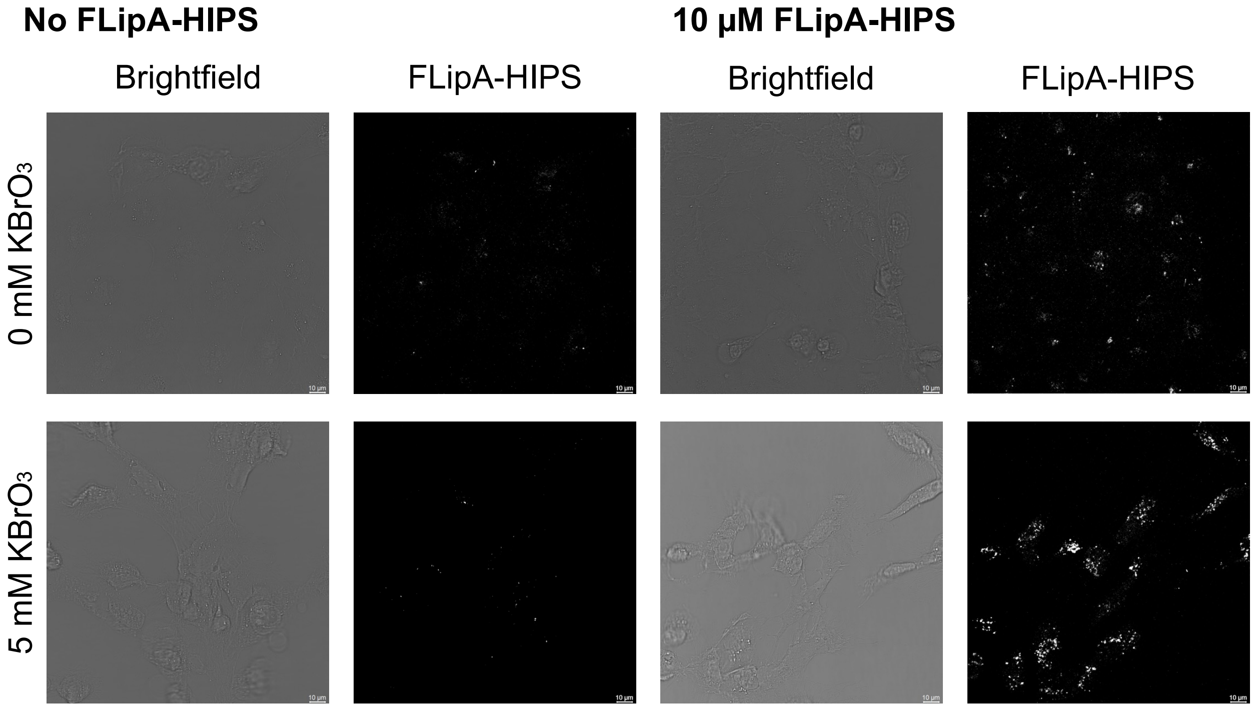

Figure S4. Fluorescence images of U2OS cells treated with or without 5 mM $KBrO_3$ for 17 h. To rule out autofluorescence of the high-energy laser (405 nm) and possibility of fluorescent adducts from $KBrO_3$ treatment alone, cells were treated with no FLipA-HIPS or 10 µM FLipA-HIPS for 4 h at 37 °C. Cells were imaged using confocal microscopy and confirmed that neither of these factors contribute to false-positive fluorescent signal. Scale bars represent 10 µm.

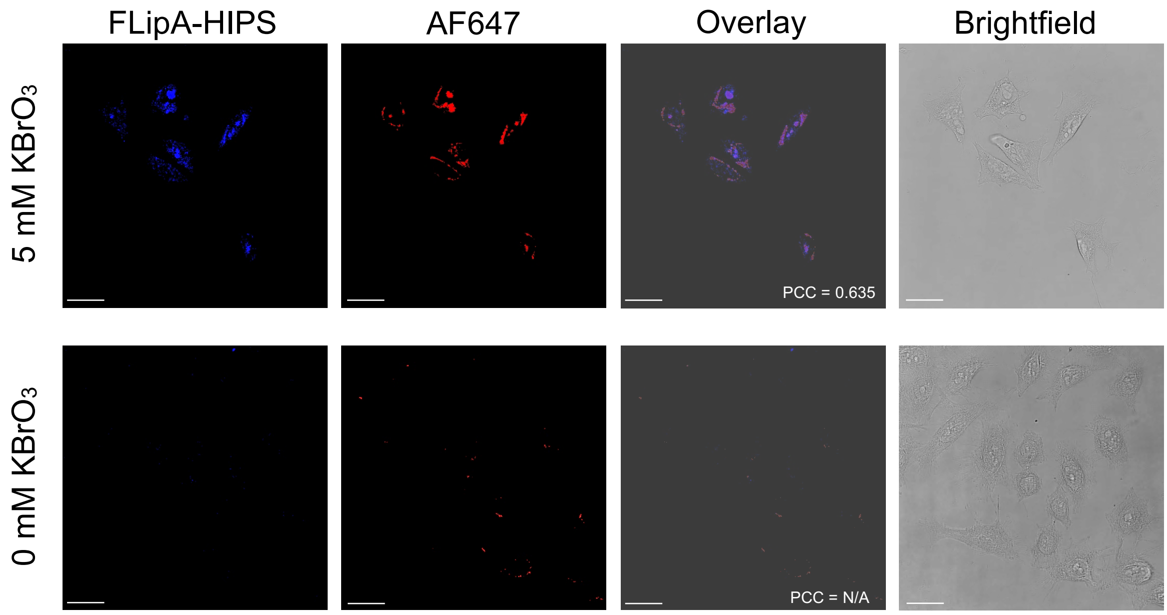

Figure S5. Fluorescence images of HeLa cells treated with or without 5 mM $KBrO_3$ for 17 h followed by 4 h of 10 µM FLipA-HIPS. Subsequent washing with PBS and fixation using cold MeOH prepared the cells of CuAAC. The cells were incubated with 2 mM THPTA, 1 mM $CuSO_4$, 10 µM azide dye (AF647) and 10 mM sodium ascorbate for 2 h at room temperature. Confocal microscopy was used to image the cells: FLipA-HIPS: λ_{ex} = 405 nm and AF647: λ_{ex} = 638 nm. Scale bars represent 25 µm.

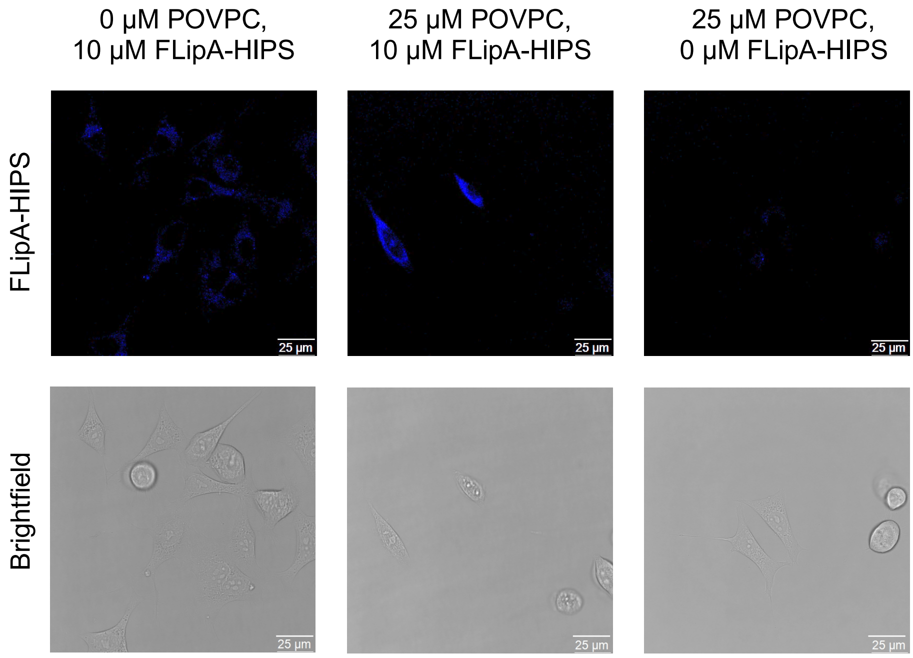

Figure S6. Fluorescence imaging of HeLa cells treated with or without 25 µM of POVPC for 1 h followed by 2 h of 10 µM FLipA-HIPS at 37 °C. Confocal microscopy used to image the cells: FLipA-HIPS excited at 405 nm. To rule out autofluorescence contributions, cells receiving POVPC, but no FLipA-HIPS were also imaged. Scale bars represent 25 µm.

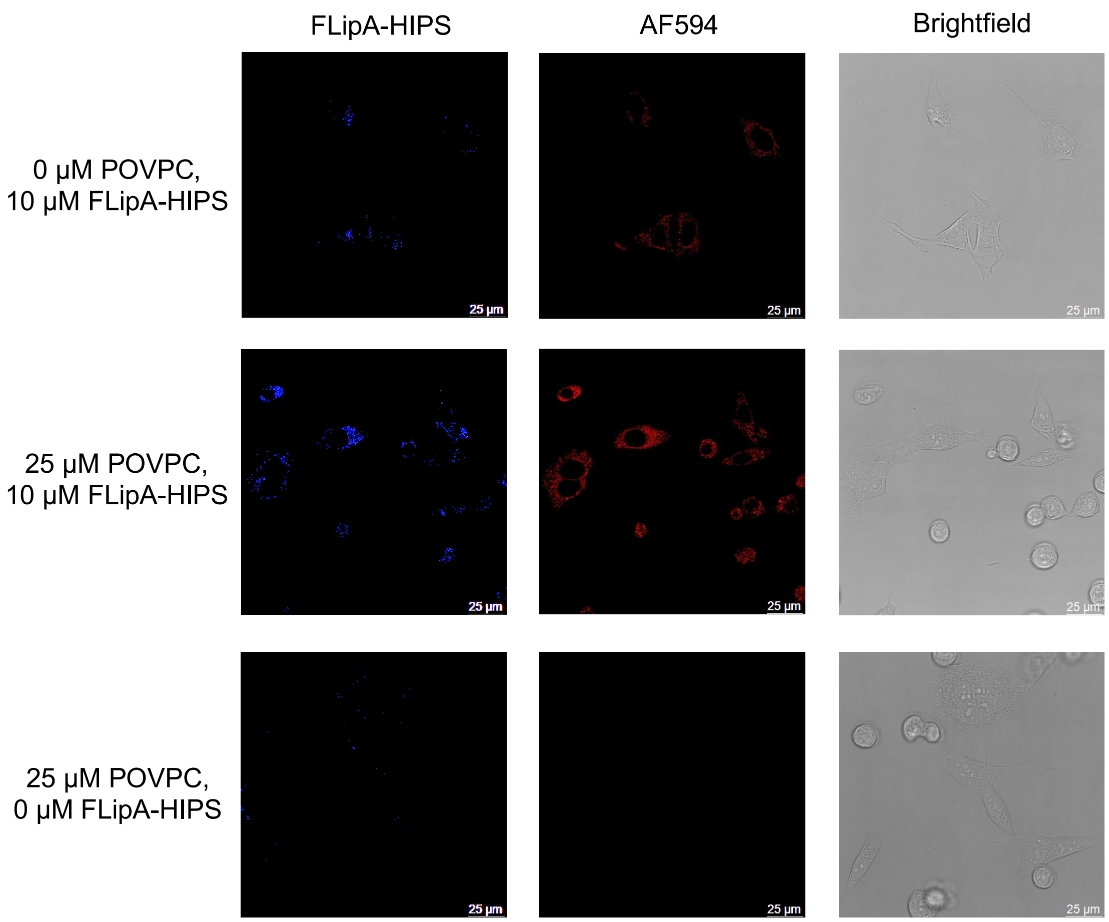

Figure S7. Fluorescence imaging of HeLa cells treated with or without 25 µM POVPC for 1 h followed by 2 h of 10 µM FLipA-HIPS. After washing with PBS, the cells were fixed using cold MeOH prepared the cells for CuAAC conditions with 2 mM THPTA, 1 mM $CuSO_4$, 10 µM azide dye (AF594) and 10 mM sodium ascorbate for 2 h at room temperature. Average PCC value of 0.778 ± 0.06 between FLipA-HIPS signal and AF594 was observed. Confocal microscopy was used to image the cells: FLipA-HIPS excited at 405 nm and AF594 excited at 561 nm.

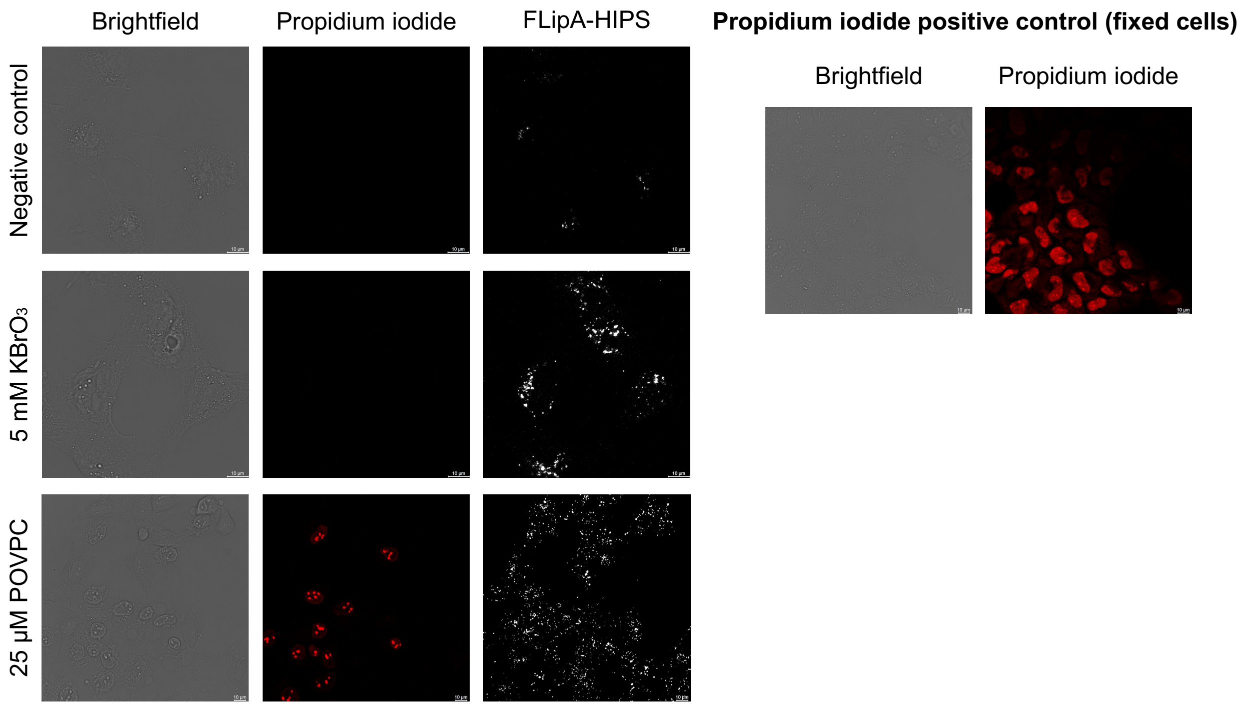

Figure S8. Fluorescence images of U2OS cells treated with 5 mM $KBrO_3$ for 17 h or 25 µM POVPC for 1 h or remained untreated. POVPC-treated cells were incubated with 10 µM FLipA-HIPS for 2 h at 37 °C while $KBrO_3$-treated cells were incubated with 10 µM FLipA-HIPS for 4 h at 37 °C. Cells were aspirated and treated with 2.5 µg/mL of Propidium Iodide for 5 min before confocal microscopy of the live cells. Cells fixed with cold MeOH and treated with 2.5 µg/mL of Propidium Iodide for 5 min were used as the positive control. Scale bars represent 10 µm. FLipA-HIPS: λ_{ex} = 405 nm and Propidium Iodide: λ_{ex} = 561 nm.

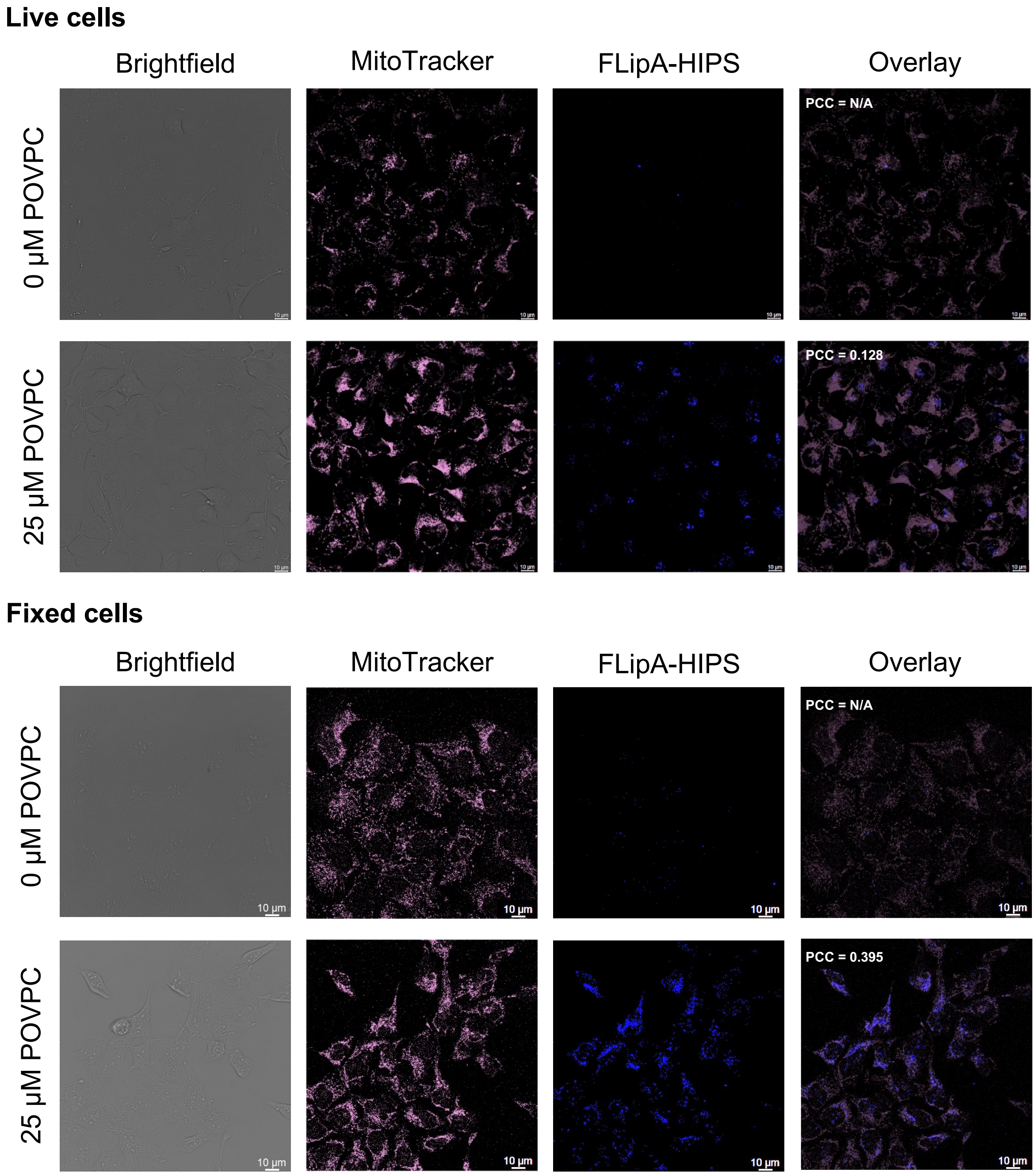

Figure S9. Fluorescence images of U2OS cells treated with or without 25 μM POVPC for 1 h followed by 2 h of 10 μM FLipA-HIPS at 37 °C. Cells were washed and treated with 300 nM of MitoTracker Red (CMXRos) for 30 min at 37 °C. Confocal microscopy was used to image the live cells. Cells were then washed with PBS and fixed using cold methanol prior to confocal microscopy imaging. Scale bars represent 10 μm. FLipA-HIPS: λ_{ex} = 405 nm and MitoTracker: λ_{ex} = 561 nm.

NMR Spectra

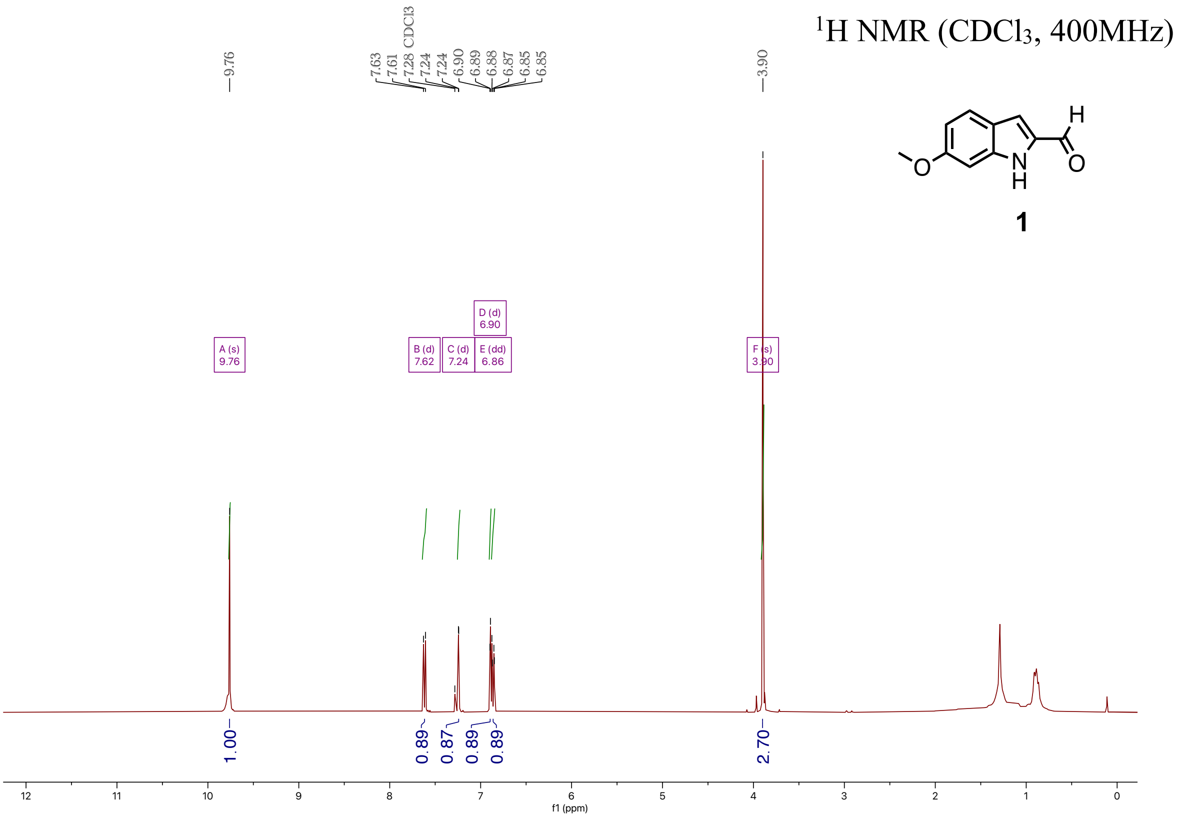
1H NMR (CDCl3, 400MHz)
1
9.76
7.63
7.61
7.28 CDCl3
7.24
7.24
6.90
6.89
6.88
6.87
6.85
6.85
3.90
A (s) 9.76
B (d) 7.62
C (d) 7.24
D (d) 6.90
E (dd) 6.86
F (s) 3.90
1.00
0.89
0.87
0.89
0.89
2.70
f1 (ppm)

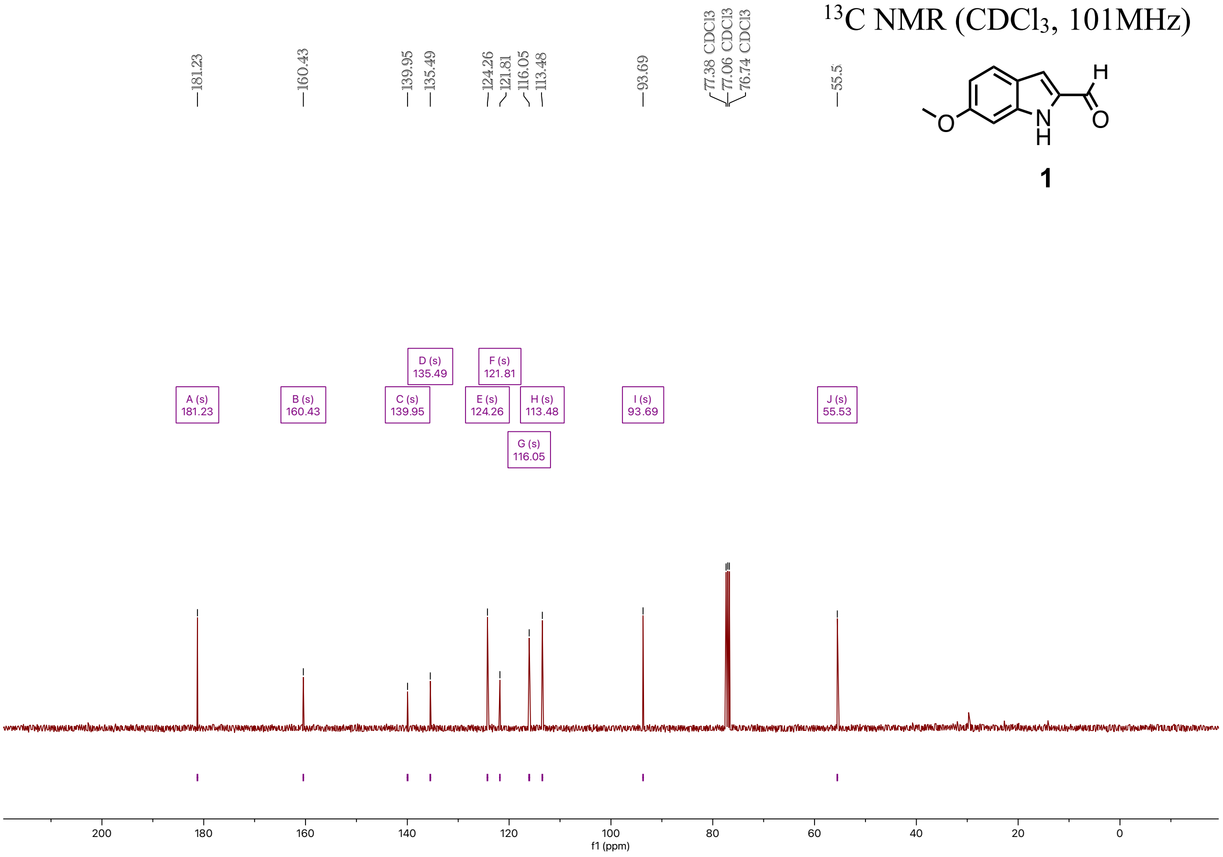
13C NMR (CDCl3, 101MHz)
1
181.23
160.43
139.95
135.49
124.26
121.81
116.05
113.48
93.69
77.38 CDCl3
77.06 CDCl3
76.74 CDCl3
55.5
A (s) 181.23
B (s) 160.43
C (s) 139.95
D (s) 135.49
E (s) 124.26
F (s) 121.81
G (s) 116.05
H (s) 113.48
I (s) 93.69
J (s) 55.53
f1 (ppm)

^{1}H NMR ($CDCl_3$, 400 MHz)

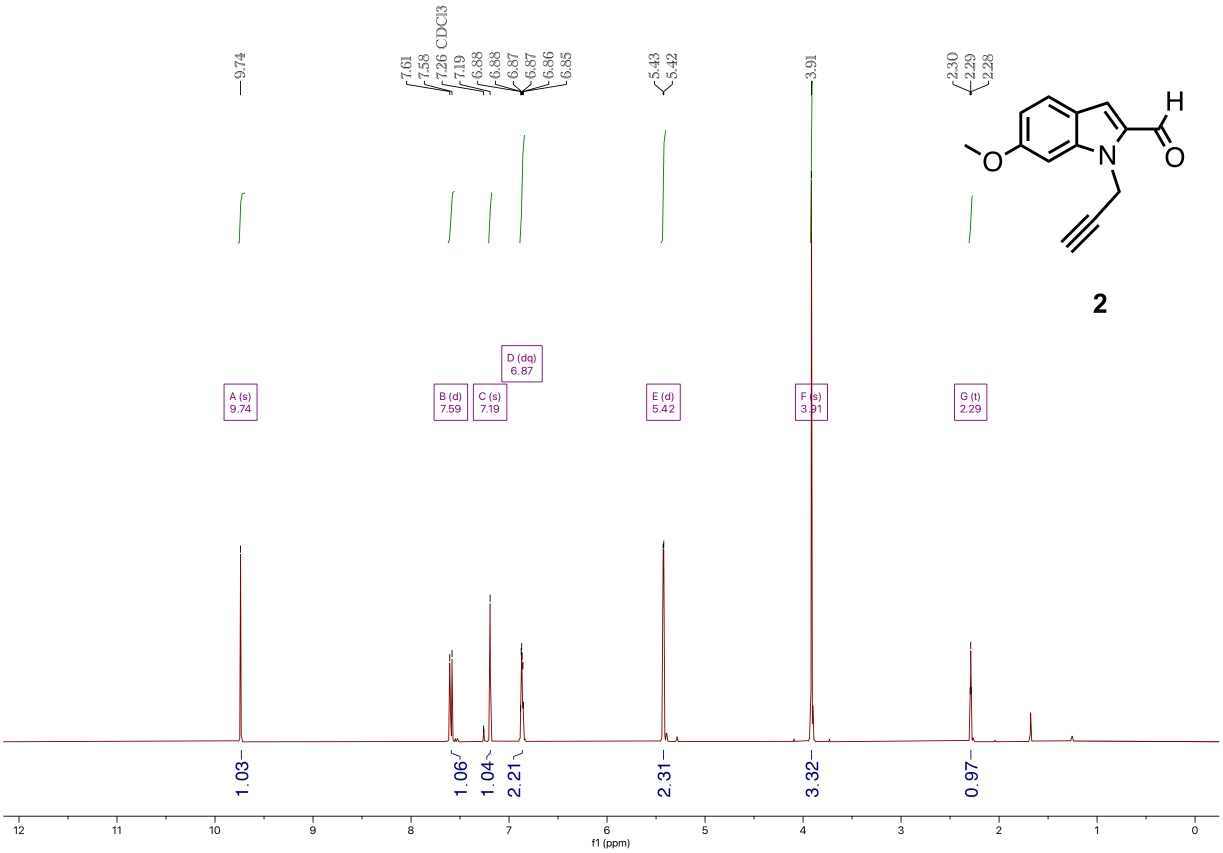

^{13}C NMR ($CDCl_3$, 101MHz)

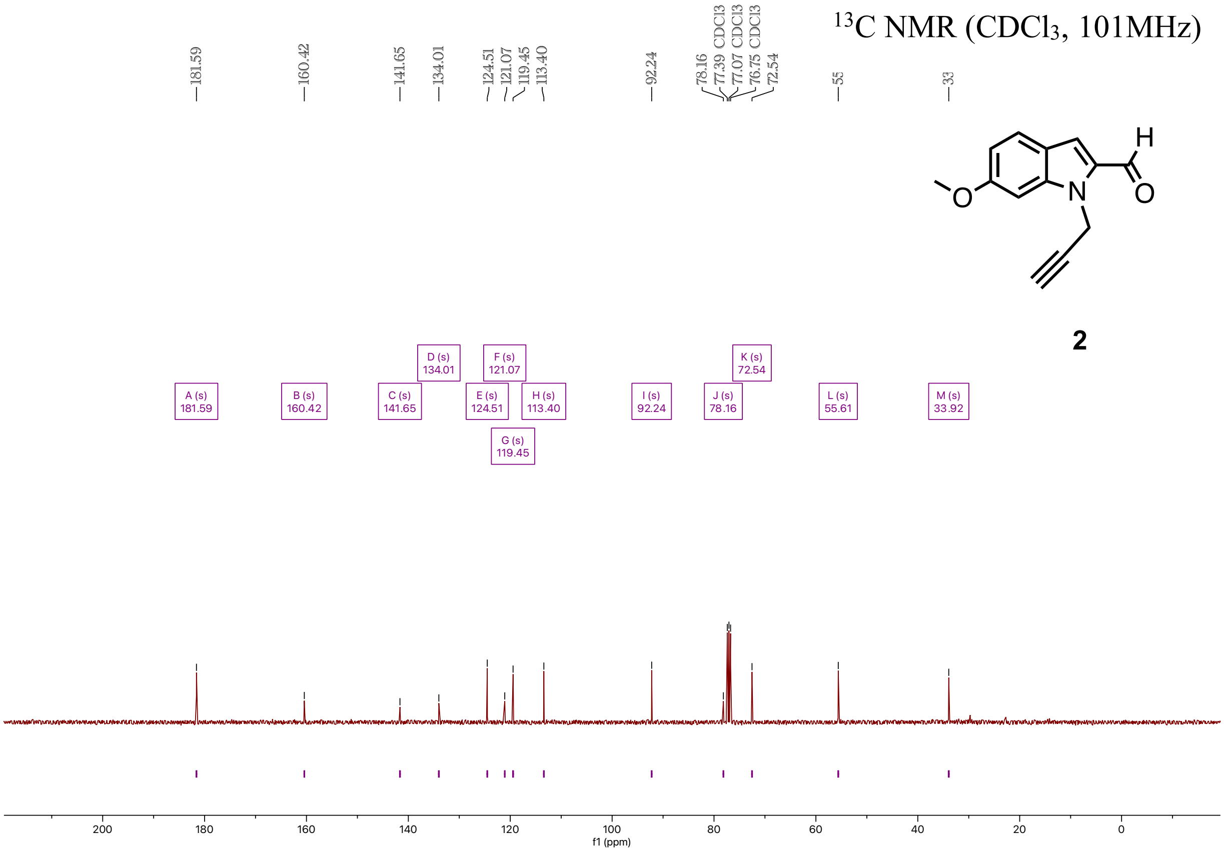

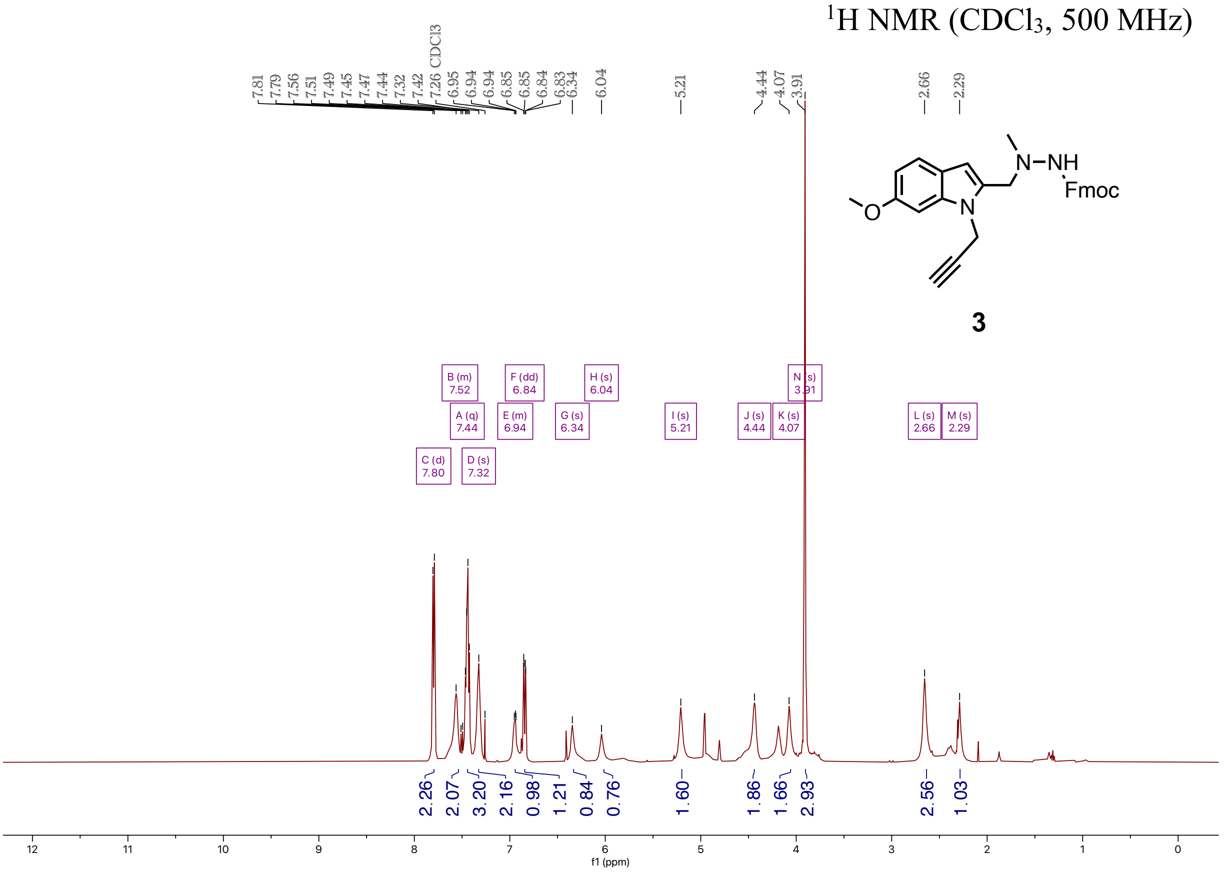

^{1}H NMR (CDCl$_3$, 500 MHz)
7.81 7.79 7.56 7.51 7.49 7.45 7.47 7.44 7.32 7.42 7.26 CDCl3 6.95 6.94 6.94 6.85 6.85 6.84 6.83 6.34 6.04 5.21 4.44 4.07 3.91 2.66 2.29
N–NH
Fmoc
O
N
3
f1 (ppm)

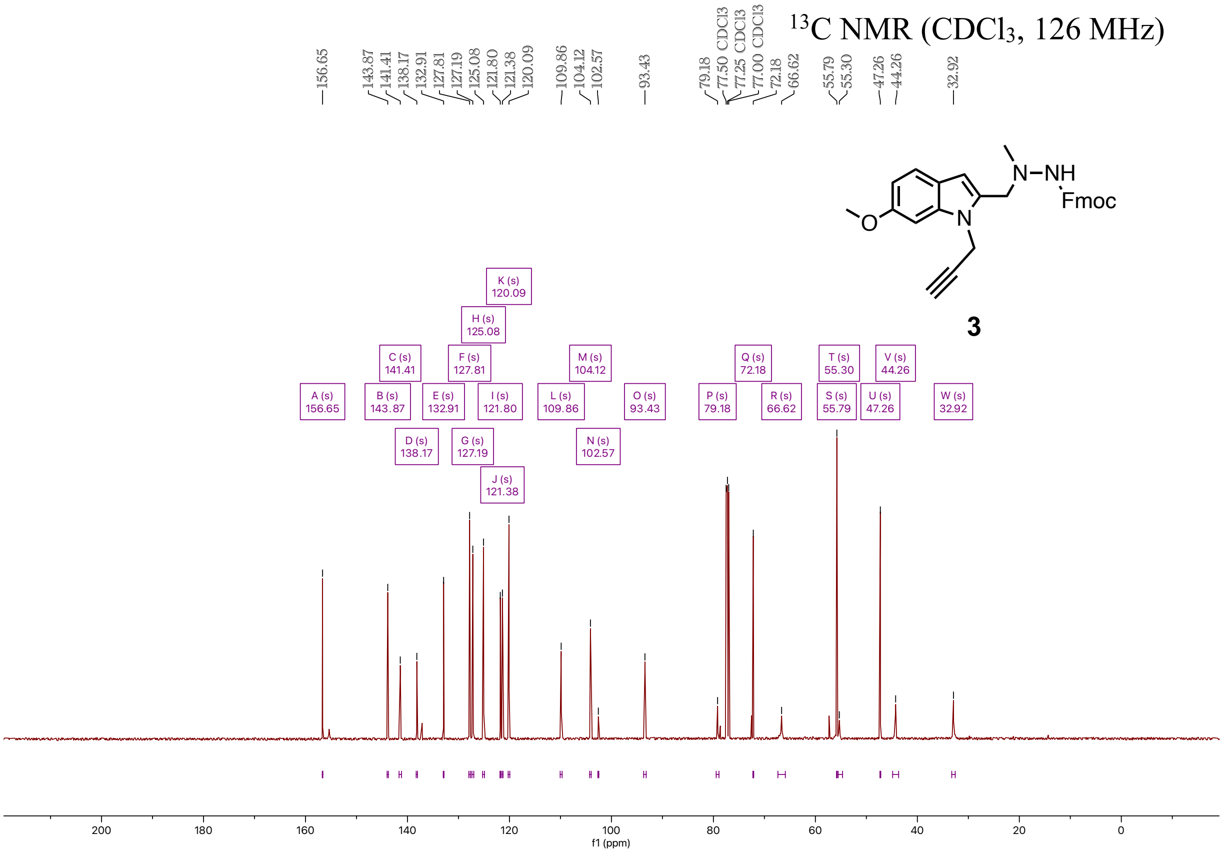

^{13}C NMR (CDCl$_3$, 126 MHz)
156.65 143.87 141.41 138.17 132.91 127.81 127.19 125.08 121.80 121.38 120.09 109.86 104.12 102.57 93.43 79.18 77.50 CDCl3 77.25 CDCl3 77.00 CDCl3 72.18 66.62 55.79 55.30 47.26 44.26 32.92
N–NH
Fmoc
O
N
3
f1 (ppm)

^{1}H NMR ($CDCl_3$, 500 MHz)

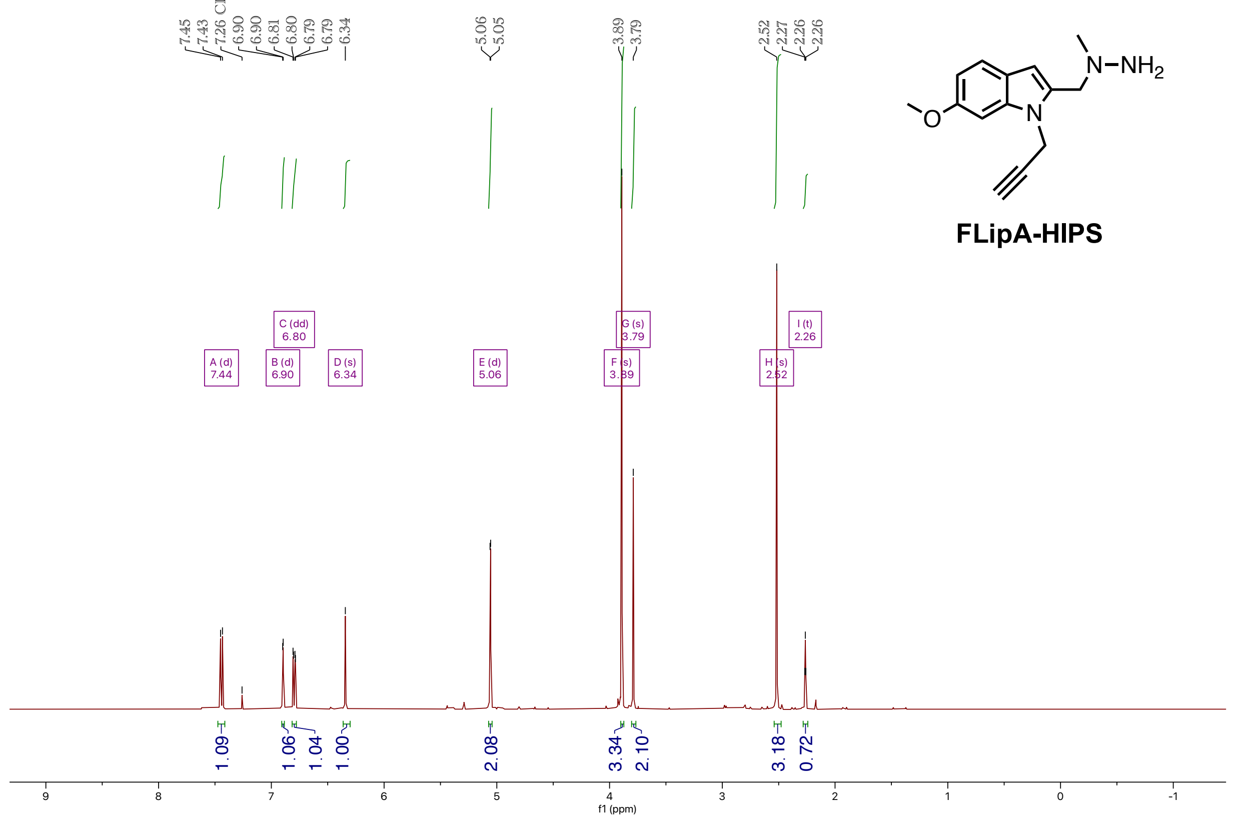

^{13}C NMR ($CDCl_3$, 126 MHz)

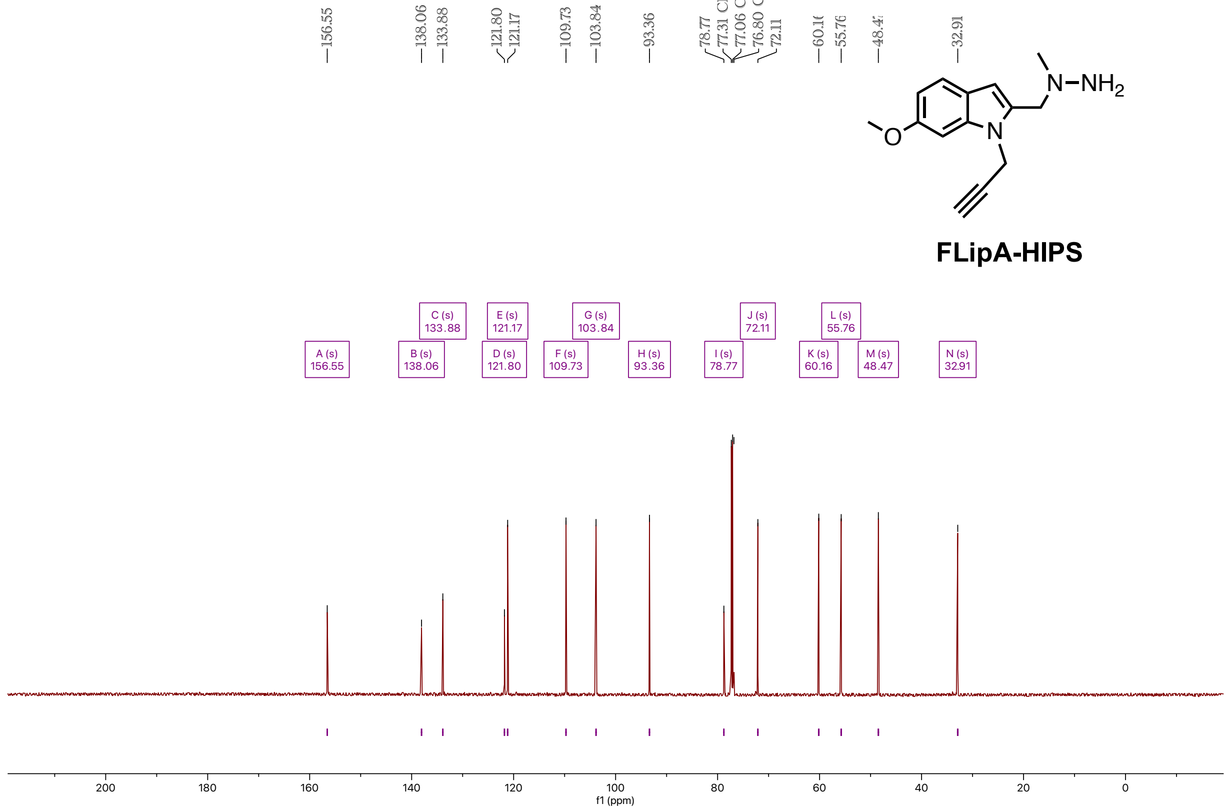

^{1}H NMR (d$_6$-DMSO, 500 MHz)

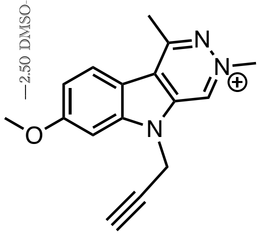

5

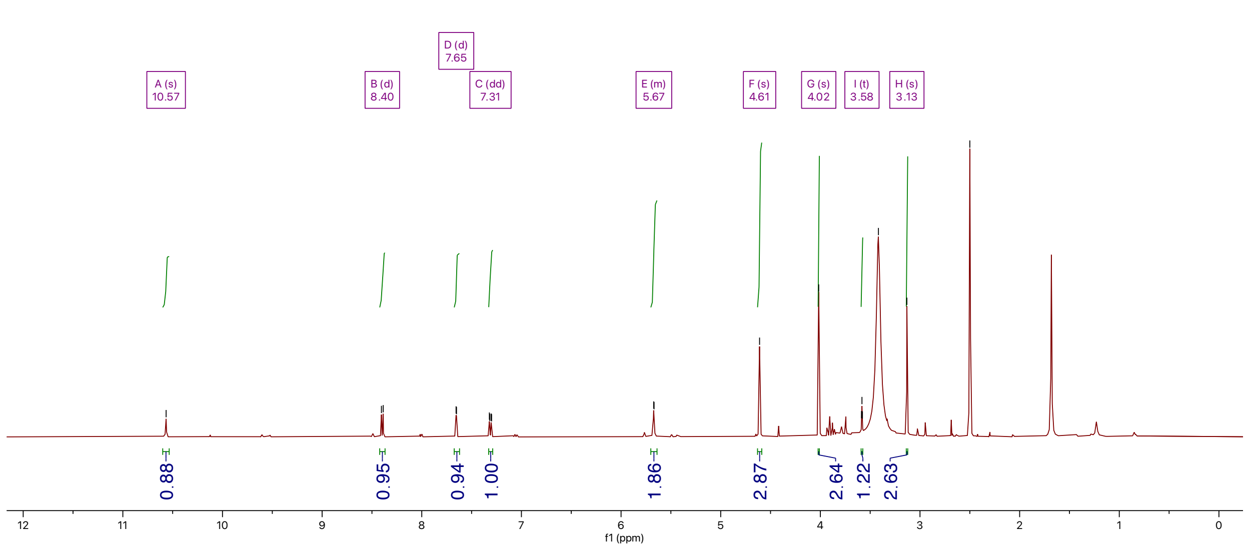

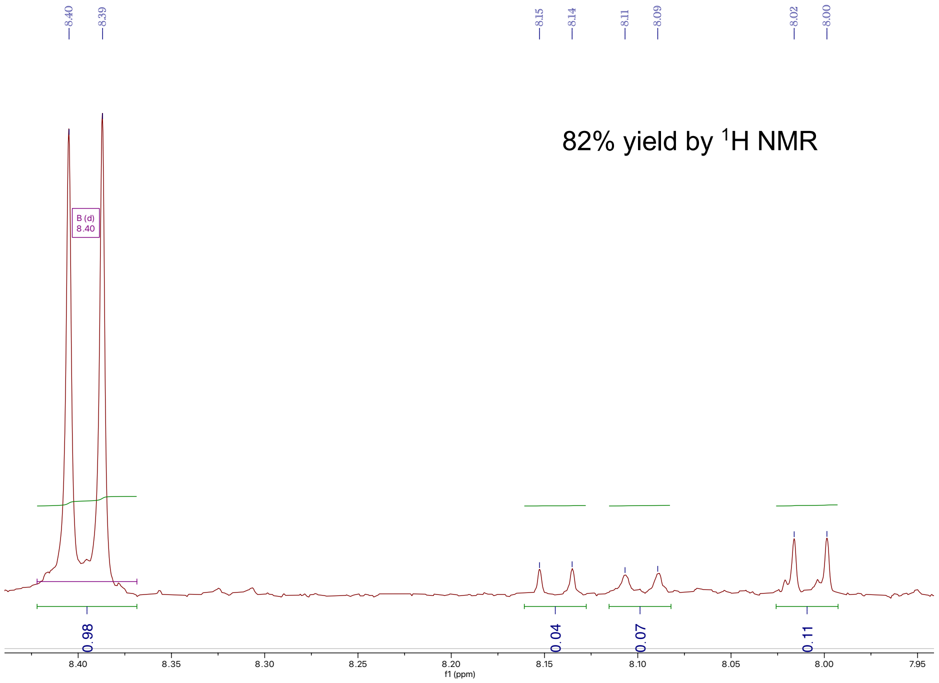

^{1}H NMR (d_6-DMSO, 500 MHz)

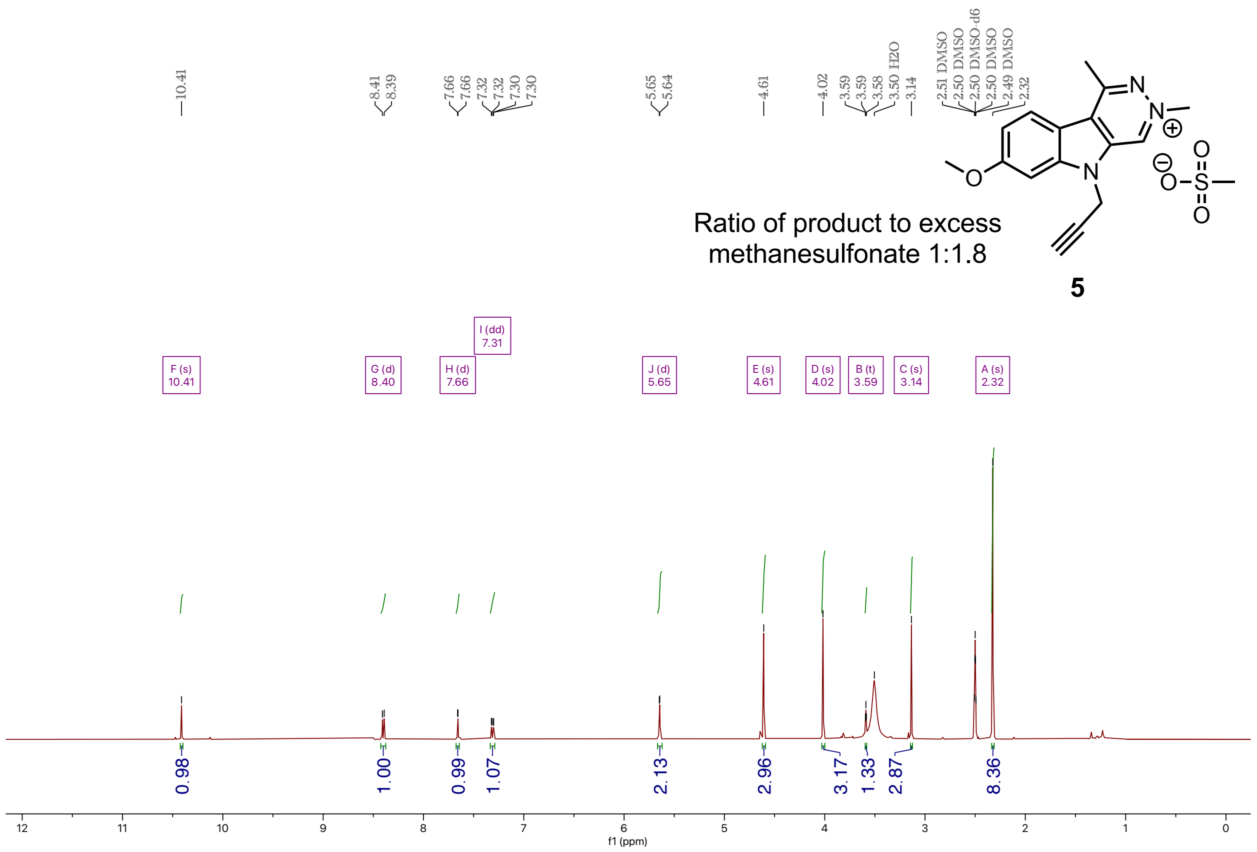

^{13}C NMR (d_6-DMSO, 126 MHz)

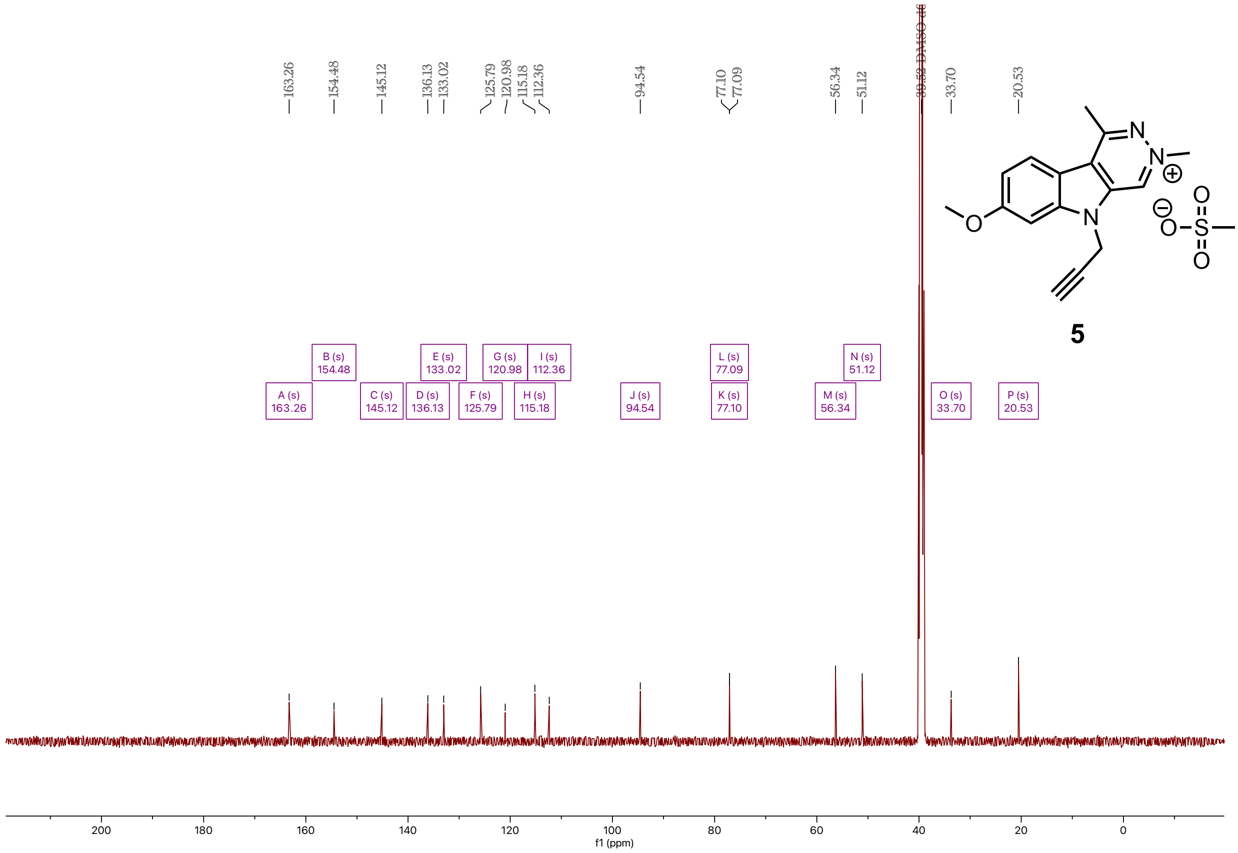

Raw Kinetic Data

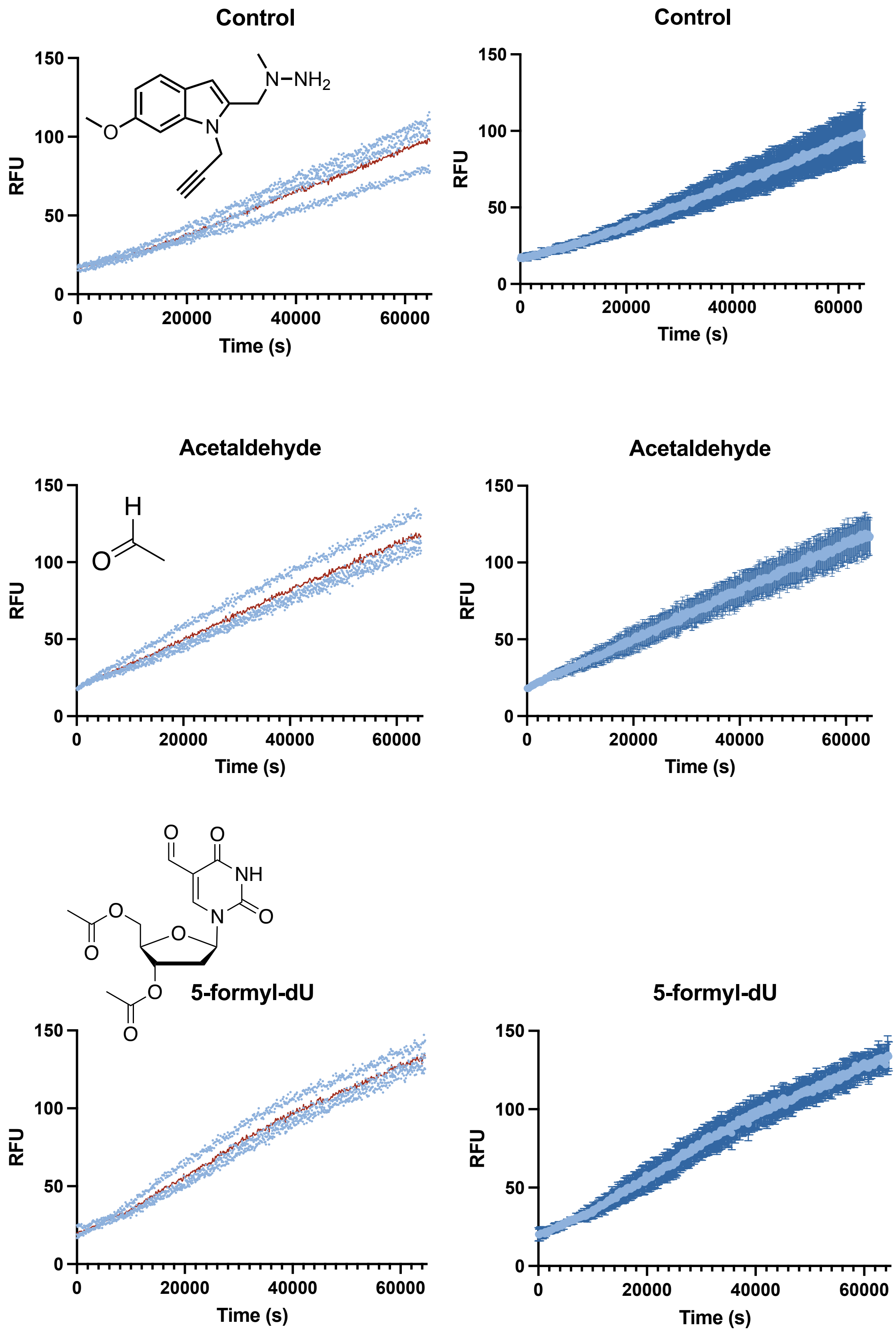

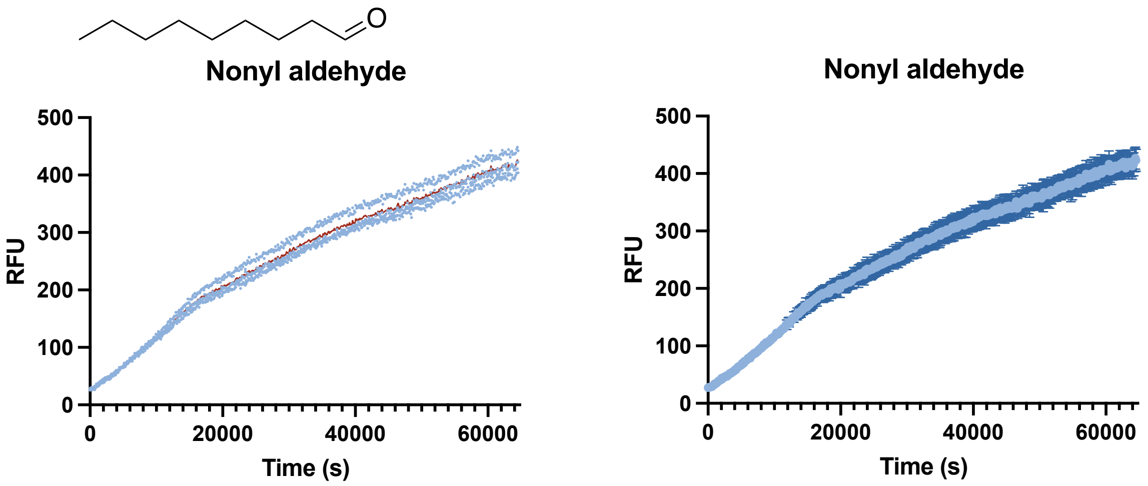
O
Nonyl aldehyde
RFU
Time (s)
Nonyl aldehyde
RFU
Time (s)

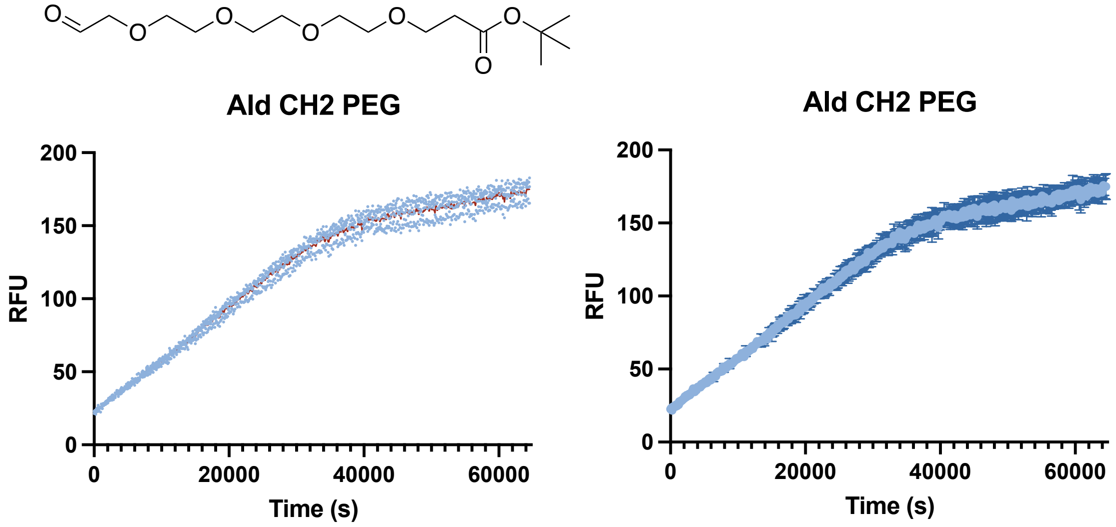
Ald CH2 PEG
RFU
Time (s)
Ald CH2 PEG
RFU
Time (s)

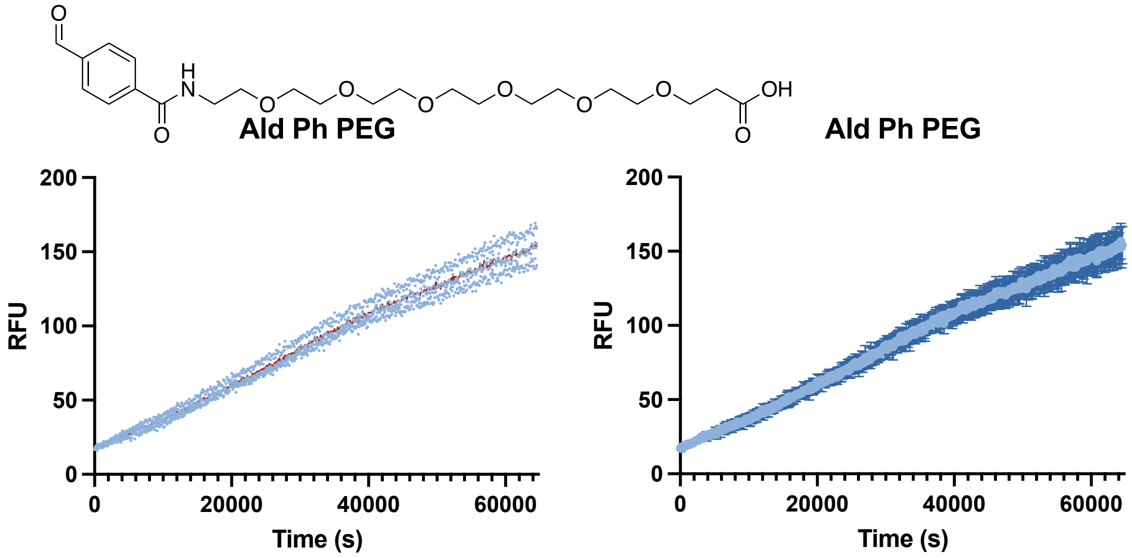
Ald Ph PEG
RFU
Time (s)
Ald Ph PEG
RFU
Time (s)

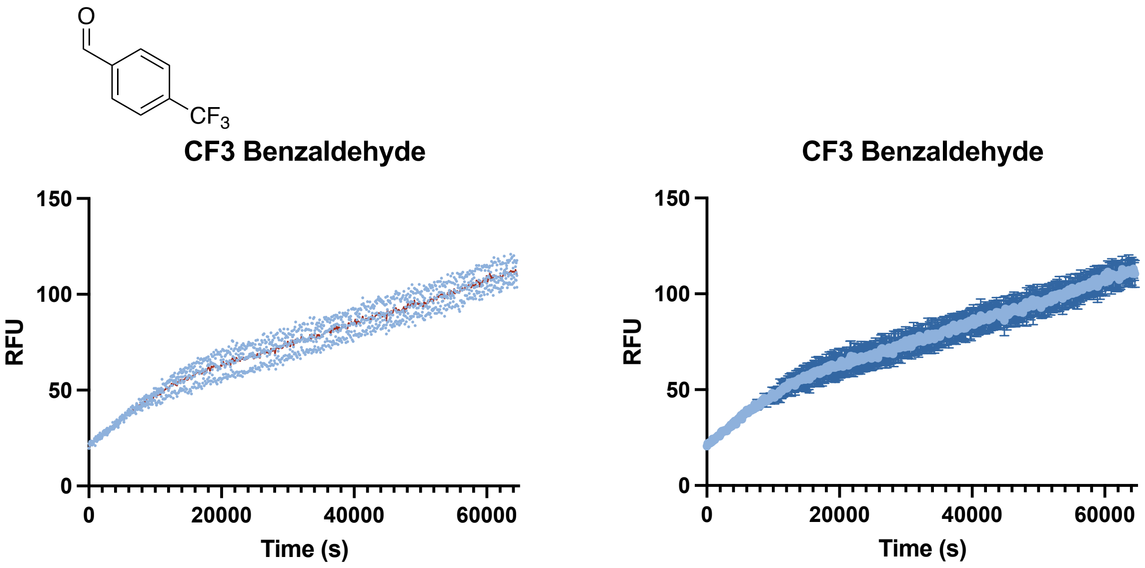

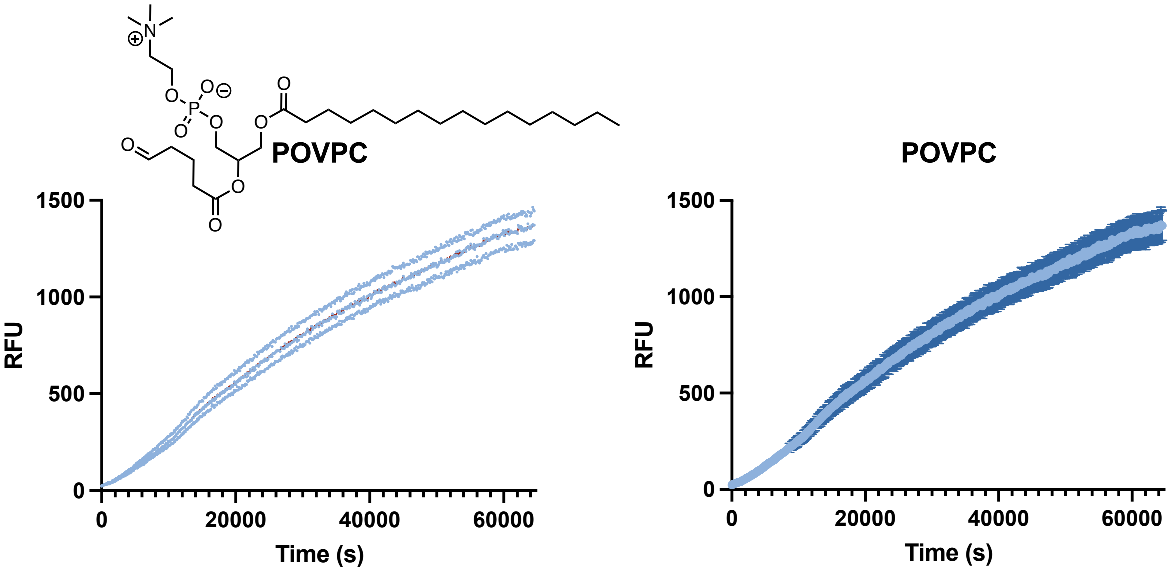

Uncorrected triplicates used to obtain the change in fluorescence and the relative kinetic analysis of FLipA-HIPS (20 μM) reacting with various aldehydes (250 μM) in 100 mM sodium citrate buffer (pH 5.0, 0.5% DMSO). The first panel for each aldehyde shows the relative fluorescence unit (RFU) increase of each triplicate in light blue with red representing the mean. The second panel shows the mean RFU with error bars.

References

1. Z. J. Liu; S. Martínez Cuesta; P. Van Delft; S. Balasubramanian, *Nat. Chem.* **2019,** *11* (7), 629-637.
2. X. Fan; X. Zhang; L. Zhou; K. A. Keith; E. R. Kern; P. F. Torrence, *J. Med. Chem.* **2006,** *49* (11), 3377-3382.
3. S. Bolte; F. P. Cordelières, *J. Microsc.* **2006,** *224* (3), 213-232.
4. C. Laummonerie; J. Mutterer Colocalization Finder. Institut de Biologie Moleculaire des Plantes: Strasbourg, France., **2004,** http://questpharma.u-strasbg.fr/html/colocalization-finder.html.
